# Midwife-led birthing centre in the humanitarian setup: An experience from the Rohingya camp, Bangladesh

**DOI:** 10.1371/journal.pgph.0004033

**Published:** 2024-12-10

**Authors:** Abdul Halim, Abu Sayeed Md. Abdullah, Fazlur Rahman, Oliva Bazirete, Sabera Turkmani, Kirsty Hughes, Sofia Castro Lopes, Andrea Nove, Mandy Forrester, Vanessa Scarf, Emily Callander, Caroline S. E. Homer

**Affiliations:** 1 Centre for Injury Prevention and Research Bangladesh (CIPRB), Dhaka, Bangladesh; 2 University of Rwanda, Kigali, Rwanda; 3 Novametrics Limited, Duffield, United Kingdom; 4 Burnet Institute, Melbourne, Victoria, Australia; 5 University of Technology Sydney, Sydney, Australia; 6 Independent consultant, Cape Town, South Africa; Health Services Academy, PAKISTAN

## Abstract

In Bangladesh, Midwife Led Birthing Centres (MLBCs) have been established to provide midwifery care and sexual and reproductive health services for the displaced Rohingya population in Cox’s Bazar. The aim of this study was to explore MLBCs in this humanitarian context from the perspectives of women, midwives, and other key stakeholders. A mixed-method case study was conducted at one of the MLBCs within the Rohingya refugee camps in Cox’s Bazar. The MLBC serves a population of approximately 8,500 people. Quantitative data were collected from the medical records and documents of the MLBC. Qualitative data included two key informant interviews (KIIs) with policy makers, one focus group discussion (FGD) with 7 midwives and ten in-depth interviews (IDIs) with Rohingya women who gave birth in this MLBC. Thematic analysis of qualitative data was performed. In 2022, 267 women gave birth at the MLBC, and 70 women with complications were transferred to higher-level facilities. Women chose the MLBC because of the respectful care provided by kind and skillful midwives, and the high-quality services. The MLBC was often recommended by community volunteers and relatives. Midwives provided a range of health services including antenatal, labour and birth, postnatal, family planning, mental health support and gender-based violence services. Challenges included language barriers, difficulty obtaining transport from home and back particularly at night in remote areas, security fears and weak cell phone coverage that affected communication for referral and follow-up. Recommendations included increased support and security staff, establishing a referral hospital nearer to the camp, refresher training for midwives and monitoring, and mentoring to improve service quality. The MLBC in the Rohingya camp shows that respectful midwifery care including management and referral of obstetric complications with wider sexual and reproductive health services can be provided in a humanitarian setting to optimize maternal and neonatal health outcomes.

## Background

The Rohingya refugee crisis has led to a massive influx of people from Myanmar into Bangladesh [1]. Since 2017, hundreds of thousands of Rohingya people have sought shelter in overcrowded and resource-constrained refugee camps in Cox’s Bazar, Bangladesh [2]. This humanitarian emergency has presented significant challenges in providing essential services, including healthcare, to the displaced population. The provision of healthcare services in refugee camps is often managed by various international organizations, non-governmental organizations (NGOs), and the host country’s government [3].

Within the refugee camps, maternal and neonatal health is a critical concern. Pregnant women and newborns face elevated risks due to the lack of accessible and safe birthing facilities, skilled healthcare providers, and adequate antenatal and postnatal care. Bangladesh strengthened rural health facilities with deployment of midwives in natural disaster affected areas, resulting in an improvement in the uptake of maternal and neonatal health care services and an improvement in outcomes [4]. The language barrier is one of the major challenges to providing services in the Rohingya camps and transportation and communication barriers are also common [5,6].

The Rohingya response authority established about 74 health facilities within the Rohingya Camps to provide a wide range of health services including maternal, neonatal, and child health care, family planning, post abortion care, treatment of sexually transmitted infections (STIs), and prevention and counselling of women and girls experiencing gender-based violence. These services are provided by doctors, nurses, midwives and paramedics. Among all facilities, 35 were deployed with 4–6 midwives, of whom one midwife works as the lead to provide a midwifery model of care including care during labour and birth 24 hours a day, 7 days a week. These facilities are known as Midwife Led Birthing Centres (MLBCs) [7]. In an MLBC, the lead healthcare professional is a midwife. The MLBC ensures care for women during normal labour and birth, access to emergency care, and is fully integrated within the healthcare system. A medical doctor is also available in each MLBC to provide consultations in cases of medical emergencies [8]. There are about 400 midwives working in 35 MLBCs [9]. By supporting women in pregnancy, normal childbirth, and referral of obstetric complications, it is hoped that the MLBCs can contribute to reducing maternal and neonatal mortality and improving overall health outcomes in this humanitarian context [10]. MLBCs are seen as an effective evidence-based intervention to address maternal and newborn health needs in these vulnerable and displaced populations [11].

The MLBCs in Cox’s Bazar have been established for four years. It was therefore decided to evaluate an MLBC to understand the enablers, barriers, facilitators and challenges in program implementation. This study provides insights for future humanitarian efforts in improving reproductive health services through MLBCs. The aim of this study was to explore MLBCs in the humanitarian context of Cox’s Bazar from the perspectives of women, midwives, and other key stakeholders, especially focusing on the experiences of giving and receiving care and identifying the enablers and challenges to the provision of such services.

## Methods

### Ethics statement

Ethical permission for the study was provided by CIPRB Ethical Review Board, Bangladesh (CIPRB/ERC/2022/11(1 Sept 2022)) and Refugee Relief and Repatriation Commissioner (RRRC) [RRRC-No.51.04.2200.010.33.030.2021–5008 (1 Nov 2022)]. Formal written consent was received from all respondents before starting the interviews and focus group discussion.

### Methodology

A mixed-method case study design was undertaken in one of the MLBCs established in the Rohingya refugee camps at Cox’s Bazar in Bangladesh. The MLBC is known as Camp 4 extension and serves a population of about 8,500 people [12]. The MLBC is supported by RTM-International (a non-government organisation) [13]. At the time of the study, the MLBC was staffed by seven professional midwives as primary health care providers, one medically trained doctor and support staff including a night guard, security officer, cleaner and administrative persons. The midwives were responsible for managing the MLBC as well as providing antenatal, childbirth, and postnatal care, family planning counseling, and referral of women and/or newborns with complications to the doctor was responsible for providing medical advice and treatment when requested or when women with complications were referred. Ethical approval for the study was obtained from the CIPRB Ethical Review Board, Bangladesh. Permission from the Refugee Relief and Repatriation Commissioner (RRRC) Bangladesh was also received to conduct the study in the Ruhingya camp. Written informed consent from each of the participants in the study was obtained.

The study was conducted from 1 November 2022 to 31 December 2022 with both quantitative and qualitative components. The quantitative component aimed to assess the type and usage of services offered by the MLBC. Quantitative data were collected from the hospital records and analyzed numerically. The qualitative aspect explored the feasibility, acceptability, enablers and challenges and perceived outcomes of the MLBC. Two key informant interviews (KIIs) were conducted with policy makers, one focus group discussion (FGD) involving all seven midwives working in the MLBC, and 10 in-depth interviews (IDIs) were conducted with Rohingya women who had given birth in the MLBC over a two-week period in December 2022. One female midwife researcher and one female anthropologist were trained in the data collection processes.

The study followed a Networks of Care (NOC) framework, which comprises four key domains including agreement and enabling environment, operational standards, efficiency/quality/ responsibility, and learning and adaptation. Within these domains, various aspects were considered, including access, quality of care, financing, community support, referral systems, and the supply/resources of health services [14]. The NOC framework informed the selection of study site, determining data collection approaches, and guiding the initial phases of analysis [Table 1].

**Table 1:** Description of the participants in the KIIs and FGDs.

Types of Interviews	Types of Respondents	Number of Interviews
KII	Policy Makers	2
IDI	Rohingya Women	10
FGD	Midwives	1 (7 midwives)

All interviews and discussions took place in private settings, using audio recording and paper forms, facilitated by the research team. Specific guidelines in Bangla language were used for different data collection methods: IDI guideline for women, FGD guideline for midwives, KII guideline for policymakers and a consent form in Bangla language for each method.

There was a dedicated quality control team. Their role involved verifying whether the data collectors correctly engaged the appropriate participants and asked relevant questions. They assessed whether the Bangla guideline and checklist was used correctly, whether the consent form was described correctly to all respondents, and if the duration and environment for data collection was satisfactory.

After data collection, the qualitative and quantitative findings underwent analysis under the supervision of the team leader. A quantitative data collection tool was designed by the research team. After initial data collection, a series of discussions were held between the analysis team to validate the data provided. Quantitative data were analyzed and presented as numbers and proportions in tabular form. Qualitative data were subjected to thematic analysis. Audio recordings were transcribed by two anthropologists independently, and these transcriptions were cross compared for accuracy. Subsequently, the transcriptions were coded independently by two research assistants. The transcription process was carried out in Bangla and later translated into English. Through this process, major themes and subthemes emerged and were coded accordingly. The responses were sorted and categorized, and relevant excerpts from the transcripts were selected to illustrate the findings.

## Results

In total, 337 women were booked at the MLBC to give birth during 2022. Of them, 299 women (89%) gave birth at the site and another 70 women (21%) with obstetric complications were transferred to higher facilities. The women were mostly referred to a non-government hospital in Cox’s Bazar, where management of complications and caesarean section is available. In total, 38 (54%) women were referred during labour and 32 (46%) women were referred after the birth. Among the referred women, 16 (42%) had caesarean section. Of the 43 (16%) women with significant complications, 10 (23%) were referred due to postpartum haemorrhage and 19 (44%) for prolonged and/or obstructed labour (Table 2).

**Table 2:** Description of activities and birth outcomes in the MLBC from January 2022 to December 2022.

Indicator	Number (%)
Population covered by this MLBC (Camp 4 extension)	8710
Number of women booked at the MLBC during pregnancy	337
Number of births in the MLBC	299 (89%)
**Women referred to a higher-level facility**	
Number of women transferred to a higher-level facility	70 (21%)
Number of women transferred during labour	38 (54%)
Number of women transferred after the birth	32 (46%)
Number of newborns transferred to a higher facility	5 (2%)
Number of women who had caesarean section at referral centre among the referred women during labour	16 (42%)
Number of women with significant complications and referred among the referred women	43 (16%)
Postpartum haemorrhage (>600mL)	10 (23%)
Prolonged and/or obstructed labour	19 (44%)
Pre-eclampsia	11 (26%)
Eclampsia	3 (7%)
Number of maternal deaths including women transferred	0
Number of stillbirths including referrals	3 (11 per 1000 live births)
Number of early neonatal deaths including referrals	1 (4 per 1000 live births)
**Postnatal care**	
Number of women receiving postnatal care at this MLBC	178 (67%)
Average length of stay in the MLBC	24 hours

The qualitative research findings are described using several domains in the Network of Care (NOC) framework. These include the positive and enabling environment, the workforce and operational standards and the provision of high-quality care. The qualitative analysis also explored the key barriers and challenges and the lessons for the future.

### The MLBC provides a positive and enabling environment

The welcoming and supportive environment was important to the women attending the MLBC. Women felt that the MLBC provided personalized and respectful care. Women reported that the midwives provided excellent care, especially during pregnancy and labour and birth. They were kind and respectful to women and their services and counseling were of high quality that made women choose this facility. The community volunteers or their relatives initially introduced them to the MLBC, and midwives’ positive endorsement further reinforced their decision to choose this location for childbirth.

During the interviews, women appreciated the midwives’ conduct for influencing their choices, for example:

*“There are no male providers in the hospital [MLBC]. All the service providers are women here- that made me feel comfortable*. *This is why I have chosen this centre for delivery”*

*“The counseling provided by the birth unit sisters [midwives] here is very good*. *They gave me respect, talked friendly as if I was part of them. Thus, I got full confidence in them and believed that I will get better care and treatment here.”*

*“I came here a few times for checkups*, *then delivered the baby*. *I like this hospital [MLBC] better than other hospitals*. *When I used to come here for an ANC checkup*, *the midwives used to talk to me very well and check me very well*.”

Women appreciated the communication style and counseling approach of the midwives. They were satisfied with the prompt service upon their arrival which was tailored to their individual needs. They reported that the presence of skilled midwives in the MLBC made them feel confident. They felt that the midwives excelled in patience and the provision of care, creating a soothing and comforting atmosphere. Women who had given birth in the MLBC said:

*“This hospital [MLBC] provides services according to the need of the patient and as soon as the patient arrives the centre. If there are any complications or need to refer, they directly provide us with the information in advance and also support us with transportation for referral ……….”-*

### The MLBC has effective workforce and operational standards

The MLBC was committed to monitoring the provision of care based on best practices to ensure quality. The staff used follow-up and tools, such as an online Google sheet or a management system, that could monitor the services and practices. The key informants highlighted the importance of such systematic monitoring in the MLBC. For example, one policy maker said:

*“We ensure the consistent delivery of quality care and services through regular follow-up of MLBC functions. For monitoring, we use an online Google sheet as a management system to monitor the frequency of midwife deliveries and their adherence to evidence-based practices.*”.

For MLBCs to function effectively there is a need for support staff like cleaners, security staff, assistants (Aya) and administrative staff. This ensures that the midwives can focus on the clinical care of women and babies and have a team around them to manage the other aspects of running an MLBC. One of the policy makers said:

*“We have one Aya and one Cleaner available in this MLBC, and they are working for the labor room, IPD, and emergency room/OPD but it is difficult for these two persons to provide all support services like cleaning, security, secretarial work etc. of this MLBC covering 24 hours. So, we need adequate support staff to run the MLBC properly”*

It was clear from the policy makers’ interviews that the midwives were serving an important role in provision of care in MLBC as explained here:

*“The MLBC provides a comprehensive range of services by midwives encompassing antenatal care, normal deliveries, postnatal care, family planning services etc. By ensuring timely availability of necessary equipment and medicines, we have the potential to enhance the quality of services provided, ultimately leading to improved healthcare outcomes.”*

### The MLBC provides quality care

The MLBC provided a range of services including antenatal, childbirth and postnatal care, family planning services, mental health support, gender-based violence (GBV) services and child maternal services (CMS). The midwives had access to a referral system that connected women to higher-level service providers when required, ensuring comprehensive care and support. The midwives also provided post-abortion care services as well as screening and treatment for STIs. The midwives collaborated with partner organizations to offer a comprehensive range of services to meet the women’s needs. The midwives emphasized the significant positive impact of the breadth of the available services on maternal and neonatal well-being in their statements, for example:

*“services like CMS and GBV, though not routine, are crucial, emphasizing the positive impact of providing services and beneficial to women”.*

*“We provide services to our pregnant mothers in a variety of ways*, *and of course*, *a midwife always works as such the care of the mother is given the highest priority”*.

The midwives were committed to service quality, that is, the use of clinical protocols, guidelines, checklist for counseling, and the visual aids for ensuring quality of services. One of the midwives explained:

*“I believe the service we offer is based on strong evidence. In our center [MLBC], we have specific clinical protocols and guidelines that I follow diligently. When needed, we also use visual aids like charts, banners, and festoons to provide counseling to mothers and conduct various types of informative sessions.”. -*

The midwives had access to a fetal heart rate doppler machine, an ultrasound machine, diagnostic laboratory equipment for biochemical testing, equipment to support birthing mothers and a radiant warmer for newborn care. Access to these technologies provided reassurance to the women and enabled the provision of a higher quality of care in the MLBC. Midwives also valued being able to access equipment like birth balls or chairs for pain relief during labour. Midwives in the focus group said:

*“Apart from regular services, we provide some other services through integrated approach, which are not regular, but this related service is provided by midwives, and I think it is positive for the women”.*

*“as a modern technology*, *we primarily use the fetoscope to listen to the heartbeat*. *If the ultrasonogram can be done*, *then we can confirm to a mother what the position of the baby is*, *which is important*!*”*.

The following statement from one woman beneficiary further emphasized on the quality of care provided by the MLBC.

*“I heard good [things] about this hospital from a community volunteer. My neighbours also agreed that this hospital [MLBC] is much better than other hospitals for checkup, tests, and delivery. So, I came here and understood it is good for mothers’ care.”–one woman who received care said*

### Lessons to improve MLBCs for the future

The study also explored the lessons to improve MLBCs in the future. All the participants emphasized the value of deploying midwives in humanitarian settings, especially within an MLBC. Participants highlighted the need for a referral hospital to service this MLBC other nearby MLBCs. During the focus group, one midwife stated.

“*It would be better if there was another hospital here where all the services would be available without referring to the emergency. And it would be nice to have an additional ultrasound machine because one machine is not enough here. And having a separate room for breastfeeding would be convenient.”*

The dedication of midwives to their roles and responsibilities was evident. Midwives highlighted the need for capacity building through training, especially in refresher programs, and also managing complex situations, such as caring for small and sick newborns and addressing complications including breech births. Midwives explained:

*“It is a great concern to manage critical cases like breech births and pre-eclampsia without readily available doctors, emphasizing the value of enhanced skills and critical thinking”.*

*“Though the midwives are experts, but additional training*, *particularly in managing prolonged labour and specific techniques like vacuum and aligning with guidelines is very important”*.

*“Further training and refresher training is necessary to improve the knowledge and skills of midwives already deployed at MLBCs*.*”*.

There are a number of barriers and challenges that need to be addressed to improve the MLBCs in the future. Language was often a barrier for effective communication between midwives and the Rohingya women. Although the midwives gradually learnt a few words in the Rohingya language, it remained a challenge. The MLBC did recruit a few Rohingya women to work in the MLBC as support staff and as translators. As an example, a woman said:

*“the midwife cannot understand my language fully or often completely then I tell that to the Attendant Khala (support staff who understand Rohingya language) who then translate these to birthing sister [midwife]. It takes time and I feel not comfortable*”

Some reported that referral of women to higher-level facilities was challenging because of restrictive nature of the husband’s attitude and needing to obtain his permission. It was suggested that counselling for husbands could help to alleviate this problem. One of the midwives mentioned:

*"When we attempt to refer any complicated pregnant mother to a higher-level hospital, the attendants of the mother particularly the husband and/or her mother-in-law usually do not agree. After a lot of counselling, they might agree but the case is already severely complicated and late. In that case, we had to face many problems.”*

Transportation was also a challenge for women and families. Roads and paths are often narrow and not passable for ambulances. One midwife explained:

*"With the transportation system that we have, the vehicle does not go inside the camp through narrow Zig Zug Road. Many a times, a pregnant mother has to be carried on the shoulder or by human support, from the car or vehicle on the nearest road because they cannot enter”*

The physical layout of the MLBC was not ideal, with bathrooms being far from the labour area. For example, one woman said:

*“Everything is good in this hospital [MLBC]. But the washroom is far away. It was very difficult to go to the washroom. Delivery patients like us have to walk a long distance to use the washroom”*

There were also challenges with communication systems. Midwives reported weak cell phone coverage which impacted on the ability to arrange for consultation and referral:

*“We have a mobile phone but does not work always properly and the signal is often very weak and interrupted. We face most problem during referral to communicate to the higher centre or the patient’s family. Mobile phone access is important to follow up the women.”*

Midwives were also concerned about their own safety and that of the women. They felt they had insufficient support staff, including security persons or a night guard, especially in such a remote camp. For example, one midwife said:

*“Our MLBC has some security system, that is not enough. We sometimes feel insecure during travel from home to the MLBC and return home especially at night. Mothers often feel insecurity to come to MLBC safely as there is no vehicle support.”*

## Discussion

The aim of this study was to explore one MLBC in a humanitarian context from the perspectives of women, midwives, and other key stakeholders, especially focusing on the experiences of giving and receiving care and identifying the enablers and challenges to the provision of such services [11]. The findings shed light on the enablers, positive aspects and challenges associated with the implementation of a MLBC within this unique and complex setting.

The presence of skilled midwives who provided high-quality counseling services emerged as a crucial enabler for the MLBC. This study found that among women admitted for labour and birth, 89% births are conducted by midwives in this MLBC. Among the pregnant women booked at MLBC, 21% referred to higher facilities due to complications during and after birth through appropriate management at MLBC by midwives. There were no maternal deaths in the study year and the stillbirth and neonatal death rates were low. The experiences of women who had received care at the MLBC underscored the positive impact of these services on the decision to choose the MLBC for childbirth. The endorsement by community volunteers and other women who had received care from the MLBCs further reinforced this choice. This shows that trust-building and peer recommendations play pivotal roles in encouraging facility utilization for maternal health care [15]. The communication style and counseling approach used by midwives was highly appreciated by the women seeking care at the MLBC. Prompt and tailored services upon arrival, and the comprehensive care provided contributed to a positive experience for the women [16]. The exclusive presence of female healthcare providers was an important aspect, creating a comfortable and safe environment for the women. The impact of midwives’ patience, childcare skills, and nurturing atmosphere was evident in women’s testimonials [17].

The comprehensive range of services offered by the MLBC, including antenatal care, childbirth care, postnatal care, family planning, mental health support, and GBV services, had a positive impact on women’s experiences [18]. The MLBC’s integrated approach, coupled with collaboration with partner organizations, facilitated the provision of a wide range of services tailored to women’s needs. The emphasis on evidence-based practices and the use of technology for follow-up and monitoring highlighted a commitment to quality care [19].

Our findings are similar to studies [10,20] that address the barriers facing MLBC including security and safety, cultural and gender norms, as well as deficiencies in infrastructure and supplies. Addressing these barriers could significantly enhance the effectiveness and accessibility of MLBCs and thus positively impact pregnancy outcomes in such contexts [19,20].

Both policy makers and midwives emphasized the need for additional support staff, including to ensure a safe environment. Continuous training and capacity-building for midwives were highlighted as essential for women and babies with complications and ensuring evidence-based practices. The women expressed satisfaction with the current services but suggested incorporating additional facilities within the MLBC to avoid referrals for certain procedures. Efforts to streamline operations through technology and computerized services were also proposed [21–23].

The findings of this study had several implications for the provision of maternal and child healthcare in humanitarian setting such as Cox’s Bazar. The positive and nurturing atmosphere, and the range of comprehensive services underscore the importance of midwife-led care in ensuring the well-being of both mothers and infants. The barriers identified highlight the need for infrastructural improvements and enhanced communication networks to facilitate emergency responses [10].

The study’s scope was limited to a specific context, and the findings may not be generalizable to other settings. Additionally, the study did not extensively explore the perspectives of healthcare providers other than midwives. Another limitation was the language barriers. The interviews were conducted in Bangla rather than in the Rohingya language. Future research could explore the scalability and sustainability of MLBCs in various humanitarian contexts and investigate the integration of MLBCs into the broader healthcare system.

## Conclusion

The experience of operating an MLBC in the Rohingya camp underscores the importance of skilled midwives, evidence-based practices, and comprehensive services in promoting maternal and child health. By prioritizing maternal and neonatal health in the Rohingya camp, this MLBC contributes to the broader effort of upholding human rights and promoting the well-being of vulnerable populations in humanitarian crises. Evidence-based policies and interventions in this MLBC aimed at improving maternal and neonatal healthcare services, as has been observed in other emergency settings worldwide. The positive aspects identified provide valuable lessons for improving maternal healthcare delivery in humanitarian settings. Addressing barriers and implementing the recommendations can further enhance the quality and accessibility of care provided by MLBCs in such contexts.

## Supporting information

S1 Text Questionnaire for quantitative data collection. (XLSX)

S2 Text Inclusivity in global research questionnaire. (DOCX)

S3 Text Guideline for focus group discussion (FGD). (DOCX)

S4 Text Guideline for In-depth Interview (IDI). (DOCX)

S5 Text Guideline for Key informant Interview (KII). (DOCX)

S1 Data Qualitative data (KII-1). (DOCX)

S2 Data Qualitative data (FGD). (DOCX)

S3 Data Qualitative data (IDI-1). (DOCX)

S4 Data Qualitative data (IDI-2). (DOCX)

S5 Data Qualitative data (IDI-3). (DOCX)

S6 Data Qualitative data (IDI-4). (DOCX)

S7 Data Qualitative data (IDI-5). (DOCX)

S8 Data Qualitative data (IDI-6). (DOCX)

S9 Data Qualitative data (IDI-7). (DOCX)

S10 Data Qualitative data (IDI-8). (DOCX)

S11 Data Qualitative data (IDI-9). (DOCX)

S12 Data Qualitative data (IDI-10). (DOCX)

S13 Data Qualitative data (KII-2). (DOCX)

S14 Data Quantitative data. (XLSX)
