# Supplementary material for: Midwife-led birthing centre in the humanitarian setup: An experience from the Rohingya camp, Bangladesh
Source: PLOS Glob Public Health. 2024 Dec 10;4(12):e0004033. doi: 10.1371/journal.pgph.0004033 (PMC11630605; doi:10.1371/journal.pgph.0004033)
Supplement: S3 Text — (DOCX) [file pgph.0004033.s003.docx]

## Midwife-led birth centres in low- and middle-income countries: A case study in Bangladesh

‡dvKvm Mªyc Av‡jvPbv/ Focus Group Discussion (FGD)

m¤§wZcÎ (INFORMED CONSENT)

| **(AbyMÖnc~e©K mv¶vrKvi ïiæ Kivi Av‡M DËi`vZv‡K c‡o †kvbvb Ges mv¶vrKvi MÖn‡Yi AbygwZ wbb\|)**  Avm&mvjvgyAvjvBKzg/Av`ve,  Avgvi bvg**____________________________ \|** Avwg XvKvq Aew¯’Z †m›Uvi di BbRywi wcÖ‡fbkb A¨vÛ wimvP©, evsjv‡`k (wmAvBwcAviwe) bv‡g GKwU M‡elYv cÖwZôvb †_‡K G‡mwQ\| Avgiv evsjv‡`‡ki ¯^v¯’¨ I cwievi Kj¨vY gš¿Yvjq‡K gv I beRvZ‡Ki ¯^v‡¯’¨i Dbœq‡b mnvqZv KiwQ\|  wgW&IqvBd Øviv cwiPvwjZ c«me ‡mev‡K›`« ¸wji †mevmg~‡ni mnRjf¨Zv I ¸YMZ gvb Dbœq‡bi Rb¨ evsjv‡`k miKvi wgW&IqvBd Gi gva¨‡g ‡mevc«`vb wel‡q ch©v‡jvPbv Kivi cwiKíbv Ki‡Q\| D³ Kvh©µ‡gi m~Î a‡i Avwg Avcbv‡K G msµvšÍ wKQz cÖkœ Ki‡Z PvB\|  Avwg Avcbv‡K wbðqZv w`w”Q †h, GB mv¶vrKvi MÖn‡Y AskMÖnY Kivi Kvi‡Y Avcbviv †Kv‡bvSuzwKi m¤§yLxb n‡eb bv hw`I GLv‡b wKQz wKQz GKvšÍB e¨w³MZ wel‡qi Dci cÖkœ Kiv n‡e\| Avcbv‡`i bvg †Kv_vI e¨envi Kiv n‡ebv Ges Avcbv‡`i cwiPq †KD †Kvbfv‡eB Rvb‡Z cvi‡ebv\| GB mv¶vrKvi MÖn‡Y †gvUvgywUfv‡e 45 †_‡K 60 wgwb‡Ui gZ mgq jvM‡e\| GB Rix‡c AskMÖnY m¤ú~Y©iƒ‡c Avcbv‡`i B”Qvaxb, Rix‡c AskMÖnb bv Ki‡jI Avcbviv †Kv‡bvai‡bi ¶wZi m¤§yLxb n‡ebbv\| Avcbviv Avgv‡K †h †Kv‡bv cÖkœ Ki‡Z cv‡ib, wbw`©ó †Kv‡bv cÖ‡kœi Reve Avcbviv w`‡Z bv PvB‡j, bvI w`‡Z cv‡ib, GgbwK Avcbviv PvB‡j †h †Kv‡bv mgq mv¶vrKvi eÜ K‡i w`‡Z cv‡ib\|  GB Rwi‡ci wel‡q Avcbvi †Kv‡bv cÖkœ Av‡Q wK?  Avcbviv mv¶vrKvi w`‡Z ivwR Av‡Qb wK? nu¨v 1 bv 2  Avwg ¯^xKvi KiwQ †h, DËi`vZvi †h wVKvbv Dc‡i †`qv Av‡Q, mvÿvrKvi ïiy nIqvi AvM ch©šÍ Avgvi Kv‡Q ARvbv wQj\| Avwg cÖkœcÎwU †`qvi Av‡M Avk¦¯Í KiwQ †h, GwU †m›Uvi di BbRywi wcÖ‡fbkb A¨vÛ wimvP©, evsjv‡`k (wmAvBwcAviwe) Gi mvgvwRK M‡elYv wefv‡Mi wbqgvbymv‡i Ges GB M‡elYvi wbqgvejx i¶v K‡i m¤úbœ Kiv n‡q‡Q\| mv¶vrKvi PjvKvjxb †h Z_¨vejx Avgv‡K †`qv n‡q‡Q, Avwg Zv Aek¨B †Mvcb ivLe\|  mv¶vrKvi MÖnYKvixi bvg: _______________________________  mv¶vrKvi MÖnYKvixi †gvevBj bs: _______________________  mv¶vrKvi MÖnYKvixi ¯^v¶it ___________________ |
| --- |

## ‡dvKvm Mªyc Av‡jvPbv (FGD)

## DËi`vZvi Dcw¯’wZ

| **µwgK** | **bvg** | **†gvevBj bs** | **eqm** | **c`we** | **c«wZôvb** | **DËi`vZvi ¯^v¶i** |
| --- | --- | --- | --- | --- | --- | --- |
|  |  |  |  |  |  |  |
|  |  |  |  |  |  |  |
|  |  |  |  |  |  |  |
|  |  |  |  |  |  |  |
|  |  |  |  |  |  |  |
|  |  |  |  |  |  |  |
|  |  |  |  |  |  |  |
|  |  |  |  |  |  |  |
|  |  |  |  |  |  |  |
|  |  |  |  |  |  |  |
|  |  |  |  |  |  |  |
|  |  |  |  |  |  |  |

**Guideline for Focus group discussions (FGD) with Health workers**

**Introductory questions:**

1. wgW&IqvBd Øviv cwiPvwjZ GB c«me ‡mev‡K‡›`« Avcbvi `vwqZ¡ wK? KZw`b hver Avcbviv GLv‡b (‡mev‡K‡›`«) KvR Ki‡Qb? GLv‡b KvRKiv wK Avcbvi wbR¯^ cQ›` wQj bvwK Avcbv‡K GLv‡b wb‡qvM †`qv n‡qwQj?
2. GB ‡mev‡K‡›`« Avcbvi mnKg©x‡`i m¤ú‡K© ejyb! Avcbvi mv‡_ Avi ‡K ‡K KvR K‡i, Zv‡`i f~wgKv Ges `vwqZ¡i mv‡_ Avcbvi `vwqZ¡i Zyjbv K‡i ejyb, GB ‡mev‡K‡›`« g~j `vwq‡Z¡ (in charge) ‡K Av‡Qb (Ges "g~j `vwq‡Z¡ _vKv" e¨vcviUv ‡Kgb ‡mB wel‡q ejyb)?

**Key questions for group discussion:**

1. miKvi wgW&IqvBd Øviv cwiPvwjZ c«me ‡mev‡K›`«‡K Kxfv‡e mg_©b K‡i? Kxfv‡e Ab¨vb¨ ms¯’v (‡hgb GbwRI) GB c«me ‡mev‡K›`« ¸wj‡K mg_©b K‡i?
2. Avcbviv Kxfv‡e KwgDwbwU‡K GB ‡mev‡K‡›`« m¤ú…³ K‡ib? (Avcbviv Kxfv‡e KwgDwbwUi mv‡_ Z_¨ Av`vb c«`vb K‡ib)?
3. GKRb wgWIqvBd Ges Zvi K¬v‡q‡›Ui g‡a¨ GKwU Av`k© m¤úK© ‡Kgb nq/ nIqv DwPZ e‡j Avcbviv g‡b K‡ib?
4. GKRb Mf©eZx gwnjv‡K ‡Kvb welq¸wj GB c«me ‡mev‡K‡›`«i ‡mev¸wji c«wZ Av¯’v Avb‡e Ges ‡Kb Ki‡e?
5. Avcbviv wKfv‡e KwgDwbwUi wbw`©ó Pvwn`v¸wj c~iY K‡ib? (Pvwn`v c~i‡Y Avcbvi ‡Kvb c«wZeÜKZv ev m¶gZv Av‡Q Kx?) KwgDwbwUi Kv‡Q ‡mev ¸wj‡K AviI M«nY‡hvM¨ Ki‡Z Avcbviv Kx K‡ib?
6. ‡mev M«nbKvixiv mvaviYZ wKfv‡e GB ‡mevi LiP enb K‡ib (c«avbZ: A_©c«`vb c×wZ wK)? Kviv mvaviYZ ‡mev‡K‡›`«i ‡mev¸wji LiP enb Ki‡Z cv‡i? miKvi ev Ab¨vb¨ ms¯’v wK ‡mev M«nbKvix‡`i Rb¨ ‡Kvb Avw_©K mnvqZvi e¨e¯’v K‡i? (we¯ÍÍvwiZ D‡jøL Ki“b)
7. Avcbviv wKfv‡e wbwðZ K‡ib ‡h, Avcbvi ‡mev‡K‡›`« D”Pgv‡bi ‡mev c«`v‡bi Rb¨ c«‡qvRbxq mKj wRwbmcÎ I miÄvgvw`i mieivn Ges ms¯’vb i‡q‡Q?
8. wgW&IqvBd Øviv cwiPvwjZ GB c«me ‡mev‡K‡›`«i ‡mev m¤ú‡K© Avcbviv wK ai‡bi Z_¨ msM«n K‡ib? (‡K›`«xq Z_¨ e¨e¯’vcbv / ‡WUv g¨v‡bR‡g›U wm‡÷g Kxfv‡e cwiPvjbv K‡ib Ges Avcbviv Kxfv‡e Z_¨ msM«‡ni welqwU cwiPvjbv K‡ib?)
9. Avcbviv wKfv‡e Ri“ix/RwUj ‡ivMx‡`i e¨e¯’vcbv K‡ib? (Avcbviv i“Mx‡`i‡K mvaviYZ ‡Kv_vq ‡idvi K‡ib? Ab¨vb¨ ¯^v¯’¨ ‡K‡›`«i mv‡_ Avcbv‡`i ‡mev‡K‡›`« wK ai‡bi ‡hvMv‡hvM Av‡Q? Zviv Kxfv‡e ‡mLv‡b hvq?) ‡idv‡ij wm‡÷gwU fvjfv‡e KvR Ki‡j djvdj ‡Kgb nq Zv D`vniY mn ejyb|
10. GB c«me ‡mev‡K‡›`«i Kg©iZ GKRb ‡mev c«`vbKvix wnmv‡e Avcbvi Rb¨ fv‡jv jvMvi gyn~Z©¸wj Kx Kx? (Avcbviv me‡P‡q ‡ewk Kx Dc‡fvM K‡ib?) GB c«me ‡mev‡K‡›`« KvR Kivi wel‡q Avcbviv ‡Kgb Abyfe K‡ib? Avcbvi Kv‡Ri cwi‡ek cwiw¯’wZ AviI fvj Kivi Rb¨ Avcbviv fwel¨‡Z Kx cwieZ©b Ki‡Z Pvb?
11. wgW&IqvBd Øviv cwiPvwjZ c«me ‡mev‡K‡›`« KvR Kivi Rb¨ ‡mev c«`vbKvix‡`i Kx Kx `¶Zv c«‡qvRb? Avcbviv wK g‡b K‡ib GB ‡mev‡K‡›`« mg¯Í ¯^v¯’¨‡mev c«`vbKvixi c«‡qvRbxq `¶Zv i‡q‡Q? bv n‡j DbœwZi Rb¨ Kx Kx Kiv DwPZ?
12. Avcbviv Kxfv‡e wbwðZ Ki‡eb ‡h Avcbviv ‡h ‡mev c«`vb K‡ib Zv c«gvY-wfwËK ‡mev? Avcbviv wK ‡Kvb wb‡`©wkKv e¨envi K‡ib? GB wb‡`©wkKv mnvqK wnmv‡e Avi wK wK Kiv `iKvi?
13. Avcbviv hw` wgW&IqvBd Øviv cwiPvwjZ ‡mevi ¸Ygvb Ges `¶Zv DbœZ Kivi cwiKíbv K‡ib, Zvn‡j Avcbviv Kx Kx (c«avb wZbwU) civgk© ‡`‡eb?
14. Kx Kx Dcv‡q wgW&IqvBd Øviv cwiPvwjZ GB c«me ‡mev‡K‡›`«i ‡mev¸wj bvix evÜe e‡j Avcbviv g‡b K‡ib?
15. wbqwgZ ‡mev¸wj QvovI D™¢vebx Ges Abb¨ wKQy Kx Av‡Q , hv Avcbviv Avcbvi ‡mev‡K‡›`« c«`vb Ki‡Qb? GwU Kxfv‡e ‡mev M«nbKvix‡`i Ges/A_ev ¯^v¯’¨‡mev c«`vbKvix‡`i DcKvi K‡i?
16. wgW&IqvBd Øviv cwiPvwjZ ‡mevi Ask wnmv‡e Ggb ‡KvbI AvaywbK c×wZ ev c«hyw³ Av‡Q wK, hv Avcbviv e¨envi Ki‡Qb? GwU Kxfv‡e M«vnK‡`i Ges/A_ev ¯^v¯’¨‡mev c«`vbKvix‡`i DcKvi K‡i? ‡Kvb ai‡bi c«hyw³ ev cš’v fwel¨‡Z ‡mev DbœZ Ki‡e?
17. hviv GLv‡b ‡mev wb‡Z hviv mvg_©evb bq Zv‡`i Rb¨ Avcbviv Kxfv‡e wgW&IqvBd Øviv cwiPvwjZ ‡mev¸wj ‡bqvi myweav ‡`‡eb, ‡hgb n‡Z cv‡i Zviv Lywe `wi`« ‡h Zviv mn‡R hvZvqvZ Ki‡Z cv‡i bv? Avcbviv wKfv‡e fwel¨‡Z GB ‡mev DbœwZ Ki‡Z cv‡ib?
