# Supplementary material for: Midwife-led birthing centre in the humanitarian setup: An experience from the Rohingya camp, Bangladesh
Source: PLOS Glob Public Health. 2024 Dec 10;4(12):e0004033. doi: 10.1371/journal.pgph.0004033 (PMC11630605; doi:10.1371/journal.pgph.0004033)
Supplement: S4 Text — (DOCX) [file pgph.0004033.s004.docx]

## Midwife-led birth centres in low- and middle-income countries: A case study in Bangladesh

we‡kl mv¶vrKvi / In-depth Interview (IDI)

m¤§wZcÎ (INFORMED CONSENT)

| **(AbyMÖnc~e©K mv¶vrKvi ïiæ Kivi Av‡M DËi`vZv‡K c‡o †kvbvb Ges mv¶vrKvi MÖn‡Yi AbygwZ wbb\|)**  Avm&mvjvgyAvjvBKzg/Av`ve,  Avgvi bvg**____________________________ \|** Avwg XvKvq Aew¯’Z †m›Uvi di BbRywi wcÖ‡fbkb A¨vÛ wimvP©, evsjv‡`k (wmAvBwcAviwe) bv‡g GKwU M‡elYv cÖwZôvb †_‡K G‡mwQ\| Avgiv evsjv‡`‡ki ¯^v¯’¨ I cwievi Kj¨vY gš¿Yvjq‡K gv I beRvZ‡Ki ¯^v‡¯’¨i Dbœq‡b mnvqZv KiwQ\|  wgW&IqvBd Øviv cwiPvwjZ c«me ‡mev‡K›`« ¸wji †mevmg~‡ni mnRjf¨Zv I ¸YMZ gvb Dbœq‡bi Rb¨ evsjv‡`k miKvi wgW&IqvBd Gi gva¨‡g ‡mevc«`vb wel‡q ch©v‡jvPbv Kivi cwiKíbv Ki‡Q\| D³ Kvh©µ‡gi m~Î a‡i Avwg Avcbv‡K G msµvšÍ wKQz cÖkœ Ki‡Z PvB\|  Avwg Avcbv‡K wbðqZv w`w”Q †h, GB mv¶vrKvi MÖn‡Y AskMÖnY Kivi Kvi‡Y Avcbviv †Kv‡bvSuzwKi m¤§yLxb n‡eb bv hw`I GLv‡b wKQz wKQz GKvšÍB e¨w³MZ wel‡qi Dci cÖkœ Kiv n‡e\| Avcbv‡`i bvg †Kv_vI e¨envi Kiv n‡ebv Ges Avcbv‡`i cwiPq †KD †Kvbfv‡eB Rvb‡Z cvi‡ebv\| GB mv¶vrKvi MÖn‡Y †gvUvgywUfv‡e 30 †_‡K 45 wgwb‡Ui gZ mgq jvM‡e\| GB Rix‡c AskMÖnY m¤ú~Y©iƒ‡c Avcbv‡`i B”Qvaxb, Rix‡c AskMÖnb bv Ki‡jI Avcbviv †Kv‡bvai‡bi ¶wZi m¤§yLxb n‡ebbv\| Avcbviv Avgv‡K †h †Kv‡bv cÖkœ Ki‡Z cv‡ib, wbw`©ó †Kv‡bv cÖ‡kœi Reve Avcbviv w`‡Z bv PvB‡j, bvI w`‡Z cv‡ib, GgbwK Avcbviv PvB‡j †h †Kv‡bv mgq mv¶vrKvi eÜ K‡i w`‡Z cv‡ib\|  GB Rwi‡ci wel‡q Avcbvi †Kv‡bv cÖkœ Av‡Q wK?  Avcwb mv¶vrKvi w`‡Z ivwR Av‡Qb wK? nu¨v 1 bv 2  DËi`vZvi bvg: ___________________________________  DËi`vZvi †gvevBj bs: ________________________________  DËi`vZvi ¯^v¶i: _______________________  Avwg ¯^xKvi KiwQ †h, DËi`vZvi †h wVKvbv Dc‡i †`qv Av‡Q, mvÿvrKvi ïiy nIqvi AvM ch©šÍ Avgvi Kv‡Q ARvbv wQj\| Avwg cÖkœcÎwU †`qvi Av‡M Avk¦¯Í KiwQ †h, GwU †m›Uvi di BbRywi wcÖ‡fbkb A¨vÛ wimvP©, evsjv‡`k (wmAvBwcAviwe) Gi mvgvwRK M‡elYv wefv‡Mi wbqgvbymv‡i Ges GB M‡elYvi wbqgvejx i¶v K‡i m¤úbœ Kiv n‡q‡Q\| mv¶vrKvi PjvKvjxb †h Z_¨vejx Avgv‡K †`qv n‡q‡Q, Avwg Zv Aek¨B †Mvcb ivLe\|  mv¶vrKvi MÖnYKvixi bvg: _______________________________  mv¶vrKvi MÖnYKvixi †gvevBj bs: _______________________  mv¶vrKvi MÖnYKvixi ¯^v¶it ___________________ |
| --- |

**Guideline for In-depth Interview (IDI) with women**

***Introductory questions:***

1. Avcbvi mv¤ú«wZK c«me m¤ú‡K© Avgv‡K ejyb (wgW&IqvBd Øviv cwiPvwjZ GB c«me ‡mev‡K‡›`«i bvg)
2. GB c«mewU KLb I KZw`b Av‡M (ZvwiL I mgq) n‡qwQj? Avcbvi wK ‡Q‡j bvwK ‡g‡q wkï c«me n‡qwQj?
3. GUv wK Avcbvi c«_g c«me wQj? bv n‡j Av‡M ‡Kv_vq c«me Kwi‡qwQ‡jb?

***Key interview questions:***

1. Avcwb wgW&IqvBd Øviv cwiPvwjZ c«me ‡mev‡K›`« m¤ú‡K© Kxfv‡e ï‡b‡Qb Ges ‡Kb Avcwb GB ‡mev‡K›`« ‡e‡Q wb‡q‡Qb?
2. Avcwb wgW&IqvBd Øviv cwiPvwjZ c«me ‡mev‡K›`« m¤ú‡K© wK wK welq cQ›` K‡i‡Qb?
3. wgW&IqvBd Øviv cwiPvwjZ GB c«me ‡mev‡K‡›`«i Kg©x‡`i ‡Kvb welq ¸‡jv Avcwb cQ›` K‡i‡Qb? (gšÍe¨ Ki‡Z ev c«kœ wRÁvmv Ki‡Z ¯^v”Q›`¨ ‡eva K‡ib)
4. Avcbvi c«me ‡mevi wel‡q wm×všÍ ‡bËqv‡Z ‡mev c«`vbKvixMY Kxfv‡e Avcbv‡K Ges Avcbvi cwievi‡K mn‡hvwMZv K‡iwQj?
5. wgW&IqvBd Øviv cwiPvwjZ c«me ‡mev‡K›`« Kxfv‡e Avcbvi Pvwn`v‡K m¤§vb K‡i‡Q e‡j Avcwb g‡b K‡ib? (‡hgb - fvlv, Rb¥MZ Askx`vi, mvs¯‹…wZK HwZ‡n¨i c«wZ m¤§vb hv gwnjvi Rb¨ ¸i“Z¡c~Y©)
6. Avcwb c«me ‡mev‡K‡›`« ‡mev M«n‡Yi LiP wKfv‡e enb K‡i‡Qb (Avcbv‡K ‡mev M«n‡Yi LiP w`‡Z wKfv‡e ev Kviv mvnvh¨ K‡i‡Q)? (‡hgb - e¨enviKvixi wd, cwienb LiP, wb‡Ri Ges cwiev‡ii m`m¨‡`i Rb¨ Lvevi Ges evm¯’vb, Ily‡ai LiP, miÄv‡gi LiP (‡hgb m¨vwbUvwi c¨vW)
7. Avcwb wK Ab¨vb¨ gwnjv‡`i wgW&IqvBd Øviv cwiPvwjZ GB c«me ‡mev‡K‡›`« ‡mev M«n‡Y DrmvwnZ Ki‡eb? hw` n¨vu nq Z‡e ‡Kb Ki‡eb ev hw` bv nq Z‡e ‡Kb Ki‡eb bv?
8. fwel¨‡Z AviI fvj ‡mev M«n‡Yi Rb¨ ‡Kvb welq¸wj cwieZ©b Ki‡Z n‡e e‡j Avcwb g‡b K‡ib (c«avb wZbwU welq D‡jøL Kiæb)?
9. Avcwb wK g‡b K‡ib ‡h wgW&IqvBd Øviv cwiPvwjZ GB c«me ‡mev‡K‡›`« D”P gv‡bi c«meKvjxb ‡mev c«`v‡bi Rb¨ c«‡qvRbxq mg¯Í ¯^v¯’¨Kg©x, DcKiY Ges miÄvg i‡q‡Q? fwel¨‡Z GwU AviI fvj Kivi Rb¨ Avi Kx Kiv DwPZ e‡j Avcwb g‡b K‡ib?
10. wgWIqvBdiv Kx ai‡Yi ‡mev c«`vb K‡i‡Q hv‡Z Avcwb AvZ¥wek¦vmx ‡eva K‡ib? Zviv Kx Zv‡`i KvRwU mwVKfv‡e K‡i _v‡Kb?
11. Avcbvi wbivc‡` c«me ‡mev M«nY Ki‡Z Ges Avcbvi wkïi hZœ ‡bIqvi wel‡q wgWIqvBdiv Kx Kx K‡i‡Q (hv‡Z Avcwb wb‡Ri c«wZ AvZ¥wek¦vmx ‡eva K‡ib)?
12. hLb Avcwb GB c«me ‡mev‡K›`« ‡_‡K Ae¨vnwZ (QvocÎ) ‡c‡qwQ‡jb ZLb Zviv Avcbv‡K Kx Kx KvMRcÎ w`‡qwQj? ‡m¸‡jv wK Avcbv‡K eywS‡q w`‡qwQj ?
13. GB nvmcvZv‡j Avcbvi c«me Gi Av‡M Avcbv‡K Kx Kx Z_¨ w`‡qwQj (‡hgb - hw` ‡Kv‡bv RwUjZv ev Ri“wi Ae¯’v nq, Avcbv‡K nvmcvZv‡j ¯’vbvšÍi Ki‡Z n‡e)?
14. c«m‡ei mgq ev R‡b¥i wKQy¶Y c‡iB wK Avcbv‡K ev Avcbvi wkï‡K Ab¨ ‡Kv‡bv nvmcvZv‡j ¯’vbvšÍi Ki‡Z n‡qwQj? hw` nq Z‡e ‡Kb? ‡mB AwfÁZv m¤ú‡K© ejyb| Avcwb ‡Kgb Abyfe Ki‡jb?
15. Avcwb Avcbvi evwo ‡_‡K GB c«me ‡mev‡K‡›`« wKfv‡e hvZvqvZ K‡i‡Qb? Avcbvi hvZvqvZ wKfv‡e AviI mnR Kiv hvq e‡j Avcwb g‡b K‡ib?
16. Avcwb wK fwel¨‡Z Avevi GB c«me ‡mev‡K‡›`« ‡mev M«n‡Yi Rb¨ Avm‡eb, Avcbvi ‡KvbI eÜy ev AvZ¥xq‡K GB c«me ‡mev‡K‡›`« ‡mev M«n‡Yi Rb¨ mycvwik Ki‡eb? hw` nq Z‡e ‡Kb Ki‡eb?
17. ‡h ‡h c×wZ‡Z GB c«me ‡mev‡K‡›`« ‡mev¸wj AviI DbœZ Kiv ‡h‡Z cv‡i ‡m¸‡jv wK wK? Avcwb GB c«me ‡mev‡K›`« DbœwZi Rb¨ wZbwU c«avb welq eY©bv Ki“b
18. wgW&IqvBd Øviv cwiPvwjZ GB c«me ‡mev‡K‡›`« Kx Kx ¯^v¯’¨ ‡mev Av‡Q hv Øviv GwU‡K Ab¨vb¨ ¯^v¯’¨‡mev ‡K›`« †_‡K Avjv`v Kiv hvq; ‡hLv‡b gwnjviv wbivc‡` mšÍvb Rb¥ w`‡Z cv‡ib?
19. wgWIqvBdiv Kxfv‡e Avcbv‡K m¤§vb c«`k©b K‡iwQ‡jb?
20. wgWIqvBdiv Kxfv‡e Avcbv‡K c«kœ wRÁvmv K‡iwQ‡jb Ges Avcbvi hv c«‡qvRb Zv wRÁvmv Ki‡Z DrmvwnZ K‡iwQ‡jb?
21. wgWIqvBdiv Kxfv‡e Avcbv‡K Avcbvi ¯^v‡¯’¨i h‡Zœi wel‡q wb‡Ri wm×všÍ wb‡Z Dr&mvwnZ K‡i wQ‡jb ?
