# Supplementary material for: Midwife-led birthing centre in the humanitarian setup: An experience from the Rohingya camp, Bangladesh
Source: PLOS Glob Public Health. 2024 Dec 10;4(12):e0004033. doi: 10.1371/journal.pgph.0004033 (PMC11630605; doi:10.1371/journal.pgph.0004033)
Supplement: S5 Text — (DOCX) [file pgph.0004033.s005.docx]

## Midwife-led birth centres in low- and middle-income countries: A case study in Bangladesh

we‡kl mv¶vrKvi / Key Informant Interview (KII)

m¤§wZcÎ (INFORMED CONSENT)

| **(AbyMÖnc~e©K mv¶vrKvi ïiæ Kivi Av‡M DËi`vZv‡K c‡o †kvbvb Ges mv¶vrKvi MÖn‡Yi AbygwZ wbb\|)**  Avm&mvjvgyAvjvBKzg/Av`ve,  Avgvi bvg**____________________________ \|** Avwg XvKvq Aew¯’Z †m›Uvi di BbRywi wcÖ‡fbkb A¨vÛ wimvP©, evsjv‡`k (wmAvBwcAviwe) bv‡g GKwU M‡elYv cÖwZôvb †_‡K G‡mwQ\| Avgiv evsjv‡`‡ki ¯^v¯’¨ I cwievi Kj¨vY gš¿Yvjq‡K gv I beRvZ‡Ki ¯^v‡¯’¨i Dbœq‡b mnvqZv KiwQ\|  wgW&IqvBd Øviv cwiPvwjZ c«me ‡mev‡K›`« ¸wji †mevmg~‡ni mnRjf¨Zv I ¸YMZ gvb Dbœq‡bi Rb¨ evsjv‡`k miKvi wgW&IqvBd Gi gva¨‡g ‡mevc«`vb wel‡q ch©v‡jvPbv Kivi cwiKíbv Ki‡Q\| D³ Kvh©µ‡gi m~Î a‡i Avwg Avcbv‡K G msµvšÍ wKQz cÖkœ Ki‡Z PvB\|  Avwg Avcbv‡K wbðqZv w`w”Q †h, GB mv¶vrKvi MÖn‡Y AskMÖnY Kivi Kvi‡Y Avcbviv †Kv‡bvSuzwKi m¤§yLxb n‡eb bv hw`I GLv‡b wKQz wKQz GKvšÍB e¨w³MZ wel‡qi Dci cÖkœ Kiv n‡e\| Avcbv‡`i bvg †Kv_vI e¨envi Kiv n‡ebv Ges Avcbv‡`i cwiPq †KD †Kvbfv‡eB Rvb‡Z cvi‡ebv\| GB mv¶vrKvi MÖn‡Y †gvUvgywUfv‡e 30 †_‡K 45 wgwb‡Ui gZ mgq jvM‡e\| GB Rix‡c AskMÖnY m¤ú~Y©iƒ‡c Avcbv‡`i B”Qvaxb, Rix‡c AskMÖnb bv Ki‡jI Avcbviv †Kv‡bvai‡bi ¶wZi m¤§yLxb n‡ebbv\| Avcbviv Avgv‡K †h †Kv‡bv cÖkœ Ki‡Z cv‡ib, wbw`©ó †Kv‡bv cÖ‡kœi Reve Avcbviv w`‡Z bv PvB‡j, bvI w`‡Z cv‡ib, GgbwK Avcbviv PvB‡j †h †Kv‡bv mgq mv¶vrKvi eÜ K‡i w`‡Z cv‡ib\|  GB Rwi‡ci wel‡q Avcbvi †Kv‡bv cÖkœ Av‡Q wK?  Avcwb mv¶vrKvi w`‡Z ivwR Av‡Qb wK? nu¨v 1 bv 2  DËi`vZvi bvg: ___________________________________  DËi`vZvi †gvevBj bs: ________________________________  DËi`vZvi ¯^v¶i: _______________________  Avwg ¯^xKvi KiwQ †h, DËi`vZvi †h wVKvbv Dc‡i †`qv Av‡Q, mvÿvrKvi ïiy nIqvi AvM ch©šÍ Avgvi Kv‡Q ARvbv wQj\| Avwg cÖkœcÎwU †`qvi Av‡M Avk¦¯Í KiwQ †h, GwU †m›Uvi di BbRywi wcÖ‡fbkb A¨vÛ wimvP©, evsjv‡`k (wmAvBwcAviwe) Gi mvgvwRK M‡elYv wefv‡Mi wbqgvbymv‡i Ges GB M‡elYvi wbqgvejx i¶v K‡i m¤úbœ Kiv n‡q‡Q\| mv¶vrKvi PjvKvjxb †h Z_¨vejx Avgv‡K †`qv n‡q‡Q, Avwg Zv Aek¨B †Mvcb ivLe\|  mv¶vrKvi MÖnYKvixi bvg: _______________________________  mv¶vrKvi MÖnYKvixi †gvevBj bs: _______________________  mv¶vrKvi MÖnYKvixi ¯^v¶it ___________________ |
| --- |

**Guideline on key informant interviews (KIIs) with decision-makers**

***Introductory questions:***

1. Avcbvi c«wZôvb ÓwgW&IqvBd Øviv cwiPvwjZ c«me ‡meviÓ mv‡_ Kxfv‡e RwoZ, GB e¨vcv‡i Avcbvi wb‡Ri wK wK `vwqZ¡ Av‡Q?
2. ‡`‡ki wgW&IqvBd Øviv cwiPvwjZ c«me ‡mev‡K›`« ¸wji BwZnvm eY©bv Ki“b: Zviv KLb, ‡Kb Ges Kvi Øviv c«wZwôZ n‡qwQj
3. ‡`‡k we`¨gvb wewfbœ ai‡Yi wgW&IqvBd Øviv cwiPvwjZ c«me ‡mev‡K›`« ¸wji eY©bv Ki“b (Ae¯’vb, mšÍvb Rb¥`vb QvovI c«`Ë ‡mev, kvixwiK MVb, miKvix ev ‡emiKvix LvZ, Kxfv‡e Zv‡`i A_©vqb Kiv nq, Kviv GB ‡mev‡K‡›`« ‡mev wb‡Z cv‡i, c«meKvjxb hZœ QvovI wK wK ‡mev¸wj c«`vb Kiv nq)
4. wgW&IqvBd Øviv cwiPvwjZ c«me ‡mev‡K›`« Kxfv‡e evsjv‡`‡ki e…nËi ¯^v¯’¨e¨e¯’vcbvq Ae`vb ivL‡Z cv‡i e‡j Avcwb g‡b K‡ib?

***Key interview questions:***

1. miKvi wgW&IqvBd Øviv cwiPvwjZ c«me ‡mev‡K››`«‡K Kxfv‡e mg_©b K‡i? (GB ‡¶‡Î miKv‡ii Ae¯’vb Kx?)
2. Ab¨ ms¯’v¸wj (‡hgb GbwRI) wKfv‡e Ges ‡Kvb Dcv‡q wgW&IqvBd Øviv cwiPvwjZ c«me ‡mev‡K›`«¸wj‡K mg_©b K‡i?
3. wgW&IqvBd Øviv cwiPvwjZ c«me ‡mev‡K›`« ¸‡jv KwgDwbwUi mv‡_ wKfv‡e RwoZ? (Avcwb Kxfv‡e KwgDwbwUi mv‡_ Z_¨ Av`vb c«`vb K‡ib?)
4. ‡Kvb welq¸wj gwnjv‡`i GB c«me ‡mev‡K‡›`«i ‡mev¸wji c«wZ wek¦vm Ki‡Z DrmvwnZ K‡i?
5. GB c«me ‡mev‡K‡›`«i ‡mev¸wj Kxfv‡e e¨enviKvix‡`i Rb¨ mvk«qx Kiv ‡h‡Z cv‡i? (‡mev M«nbKvix‡`i Rb¨ c«avb A_©c«`v‡bi c×wZ Kx? miKvi ‡mev e¨enviKvix‡`i Rb¨ Kx Avw_©K mnvqZv c«`vb K‡i?)
6. wgW&IqvBd Øviv cwiPvwjZ c«me ‡mev‡K‡›`«i ‡mev¸wj Kxfv‡e RbM‡Yi / KwgDwbwUi Pvwn`v c~iY K‡i? (Pvwn`v ¸wj Kx Kx, ‡hgb mvgvwRK I mvs¯§…wZK c«‡qvR‡bi Pvwn`v, ¯^v¯’¨‡mev msµvšÍ Pvwn`v)
7. GB c«me ‡mev‡K›`« ¸‡jv hw` KwgDwbwUi Pvwn`v m¤ú~Y©iƒ‡c c~iY Ki‡Z n‡j ‡mUv ‡Kgb K‡i Ki‡e (gšÍe¨ Ki“b)?
8. ‡mev¸wj‡K AviI mvk«qx, M«nY‡hvM¨ Ges D‡Ïk¨ c~i‡Yi Rb¨ Rb¨ Dchy³ Ki‡Z wZbwU c«avb Kx wRwbm Kiv ‡h‡Z cv‡i e‡j g‡b K‡ib?
9. ‡idv‡ij e¨e¯’vcbv welqUv wKfv‡e KvR K‡i? GB KvRwU fvj n‡j wK jvf n‡e?
10. c«PwjZ ¯^v¯’¨ e¨e¯’vq g‡a¨ ‡idv‡ij ‡hvMv‡hv‡Mi c_¸wj Kxfv‡e m¤ú…³ Kiv hvq? GKwU Av`k© ‡idv‡ij wm‡÷g ‡Kgb n‡e? (Kxfv‡e mKj ¯Í‡ii ‡mevi mv‡_ Z_¨ ¸‡jv Av`vb c«`vb Kiv hvq ‡hvMv‡hvM Kiv hvq? Kxfv‡e ‡mev M«nYKvix‡`i ‡idv‡ij wm‡÷g m¤ú‡K© Rvbv‡bv hvq?)
11. Avcbviv ‡Kvb ‡WUv g¨v‡bR‡g›U wm‡÷‡gi gva¨‡g Z_¨ iv‡Lb Kx Ges G¸wj‡K wKfv‡e AviI kw³kvjx Kiv ‡h‡Z cv‡i?
12. wgW&IqvBd Øviv cwiPvwjZ c«me ‡mev‡K‡›`«i D”P gv‡bi ‡mev c«`v‡bi Rb¨ mieivn Ges miÄvg Kxfv‡e wbwðZ Kiv nq? mieivn Ges m¤ú` m¤ú~Y©iƒ‡c c«`vb Kiv n‡j mieivn e¨e¯’v ‡Kgb n‡Z n‡e?
13. wgW&IqvBd Øviv cwiPvwjZ c«me ‡mev‡K‡›`«i Rb¨ Av`k© Kg©x ¯Í‡ii web¨v‡mi (level of staff) eY©bv w`b (Kg©x msL¨v Ges K¨vWvi)? GwU ev¯Íevqb Ki‡Z wK wK Ki‡Z n‡e?
14. wgW&IqvBd Øviv cwiPvwjZ c«me ‡mev‡K›`«¸wji `vwq‡Z¡ ‡K _v‡Kb? (hw` wgWIqvBd ev bvm©-wgWIqvBd `vwq‡Z¡ _v‡Kb, Z‡e Rvb‡Z Pvb Zviv ‡Kvb K¨vWv‡ii wgWIqvBd ev bvm©-wgWIqvBd Ges Zviv ‡`‡k we`¨gvb Ab¨ ‡Kvb ai‡Yi wgWIqvBd ‡_‡K Kxfv‡e Avjv`v ‡m m¤ú‡K© we¯ÍÍvwiZ D‡jøL Ki“b) "`vwq‡Z¡ _vKv"(in charge) Gi `vwqZ¡ ‡Kgb? (Zviv wK ¯^vqËkvwmZ, bvwK Zviv Ea©Zb KZ©…c‡¶i Kv‡Q wi‡cvU© K‡ib?)
15. Rbej Ges AeKvVv‡gvi ‡¶‡Î Avcwb ‡Kvb wZbwU wRwbm cwieZ©b Ki‡Z n‡e e‡j g‡b K‡ib?
16. wgW&IqvBd Øviv cwiPvwjZ c«me ‡mev‡K›`« Ges Ab¨vb¨ ai‡Yi ¯^v¯’¨ myweavi g‡a¨ Kvh©Ki mgš^q Ges Askx`vwi‡Z¡i Rb¨ ‡Kvb welq¸wj ¸i“Z¡ ‡`qv `iKvi? D`vniY w`b, wKfv‡e GUvi Dbœqb Kiv ‡h‡Z cv‡i?
17. wgW&IqvBd Øviv cwiPvwjZ c«me ‡mev‡K‡›`« wgWIqvBd Ges gvZ… I beRvZ‡Ki ¯^v¯’¨ cwiPh©vi mv‡_ RwoZ Ab¨vb¨ ¯^v¯’¨ ‡ckv`vi‡`i g‡a¨ Kvh©Ki mgš^q Ges Askx`vwiZ¡ wbwðZ Kivi Rb¨ ‡Kvb welq¸wj c«fvweZ K‡i? D`vniY w`b, wKfv‡e GUvi Dbœqb Kiv ‡h‡Z cv‡i?
18. wgW&IqvBd Øviv cwiPvwjZ c«me ‡mev‡K‡›`« KvR Kivi Rb¨ ‡mev c«`vbKvix‡`i Kx Kx `¶Zv _vK‡Z n‡e? GwU ev¯Íevqb Ki‡Z wK wK Ki‡Z n‡e?
19. wgW&IqvBd Øviv cwiPvwjZ c«me ‡mev‡K‡›`«i g‡a¨ c«`Ë ‡mev¸wj Kx Dcv‡q c«gvY-wfwËK (evidence based) Kxbv Zv Avcwb Kxfv‡e wbwðZ Ki‡eb? (‡Kvb wb‡`©wkKv ev ÷¨vÛvW© Acv‡iwUs c×wZ (SOP) wK we`¨gvb Av‡Q? (hw` _v‡K Z‡e mg¯Í ‡mevc«`vbKvixiv wb‡`©wkKv AbymiY K‡i wKbv Zv Avcwb wKfv‡e Rvb‡eb?)
20. wgW&IqvBd Øviv cwiPvwjZ c«me ‡mev‡K‡›`« wK Dcv‡q ‡mev c«`vb Kiv nq? ‡mev‡K‡›`« wK wK DbœZ gv‡bi ‡mev ‡`qv nq, Ges KLb ‡`qv nq?
21. wgW&IqvBd Øviv cwiPvwjZ c«me ‡mevi gvb Kxfv‡e g~j¨vqb Kiv nq Ges Kxfv‡e Z`viwK Kiv nq? (‡WUv Ges ‡WUv g¨v‡bR‡g›U wm‡÷‡gi gvb wKfv‡e Z`viwK Kiv nq?)
22. Avcwb hw` wgW&IqvBd Øviv cwiPvwjZ ‡mevi ¸Y¸Zgvb Ges `¶Zv DbœZ Kivi cwiKíbv K‡ib, Zvn‡j Avcwb Kx Kx Ki‡eb (wZbwU c«avb KvR)?
23. G‡`‡k wgW&IqvBd Øviv cwiPvwjZ c«me ‡mev‡K‡›`«i wZbwU fvj w`K Kx Kx? (‡mev¸wji g‡a¨ bZybZ¡ Ges wfbœZv Avb‡Z Avcwb wK wK c`‡¶c wb‡Z Pvb?)
24. wgW&IqvBd Øviv cwiPvwjZ ‡mevi Ask wn‡m‡e ‡Kvb ‡Kvb AvaywbK cš’v ev c«hyw³ e¨envi Kiv nq? GB ¸jv wKfv‡e c«‡qvRbxq I Dc‡hvMx Kiv ‡h‡Z cv‡i, D`vniY w`b
