# Supplementary material for: Midwife-led birthing centre in the humanitarian setup: An experience from the Rohingya camp, Bangladesh
Source: PLOS Glob Public Health. 2024 Dec 10;4(12):e0004033. doi: 10.1371/journal.pgph.0004033 (PMC11630605; doi:10.1371/journal.pgph.0004033)
Supplement: S1 Data — (DOCX) [file pgph.0004033.s006.docx]

***KII_Program Manager_Rtmi_ Rohingya Camp_Cox’sbazar***

***Introductory questions:***

**Q1: Explore how the respondent’s organisation is involved in MLCs, the respondent’s own role**

**Answer:** Our organization is basically midwifery. All types of maternity services are provided by the best midwives, such as antenatal care, normal or general delivery, postnatal care, family planning, and GVB services. Moreover, if we get any complicated case, we refer it to PSC Vimong to Simong. In this way, it is my responsibility to see whether midwives are serving patients through evidence-based care. And they do that and try, and that's what I'm doing.

**Q2: Describe the history of MLCs in the country: when they were established, why and by whom**

**Answer:** I've known about the midwives in this country since 2016. I started working with midwives in 2016 and continued in 2017. From 2016 until 2022, when I started working with midwives, they had formally recruited three batches. That's all I know about it.

**Q3: Describe the different types of MLC that exist in the country (probe for: location, services offered in addition to childbirth, physical design/layout, public or private sector, how they are funded, who can access care at an MLC, what services are provided in addition to childbirth care)**

**Answer:** Midwives in all parts of the country provide more than just delivery services. Apart from giving birth, they also provide family planning, mental health services, GVB services or CMS services, and cervical screening services, which are funded by the government as well as UNFPA. And the methods of postnatal family planning are also being provided by them.

**Q4: How do MLCs fit within the wider health system?**

**Answer:** The maternal death rate in these facilities, which had been a cause for worry among the community, has been stabilized by the midwives since their arrival, and we expect that this trend will continue in the future. In these centers, midwives not only assist with deliveries, but we also anticipate that they will help to further lower the rate of maternal mortality.

***Key interview questions:***

**Q5: How does the government support the MLCs? (What is the position of government in relation to MLCs?)**

**Answer:** What could be seen is that there were no midwives in various UFWCs or sub-centers; now, midwives are being appointed in every district and upazila of Bangladesh. Our midwives are doing how to reduce maternal mortality rates and ensure family planning methods at every community level. By doing this, the government is fully helping us.

**Q6: Which other organisations (e.g. NGOs) support the MLCs and in what ways?**

**Answer:** Other organizations, such as NGOs, engage with midwives to ensure proper and high-quality service delivery at the delivery centers. While not all of them deal with SRS or sexual activity, the majority of them do so with assistance from the government, which receives funding from UNFPA.

**Q7: How is the community engaged or involved? (How do you get buy-in from community?)**

**Answer:** In our community, there are always some community health workers who go to the field and make people aware of all kinds of services, and if we feel the need, we also go. Every time midwives go to the field, they collaborate with them to provide awareness of all kinds of services.

**Q8: What factors encourage women to trust the MLC services?**

**Answer:** Like every other center or facility, our midwives are friendly to women. Additionally, adolescents feel at ease visiting this facility. This encourages women to visit and have faith in the midwives, whether they are pregnant mothers or family planning clients. They also regularly come to the facility for various forms of follow-up.

**Q9: How are MLC services made affordable for the users? (What is the main payment method for service users? What financial support does the government provide for service users?)**

**Answer:** As we provide all our services in government facilities, we do not take any money from our clients or patients.

**Q10: How do the MLC services fulfil the community’s needs? (probe for social and cultural needs as well as clinical needs)**

**Answer:** The services that midwives usually provide are antenatal care, delivery, postnatal care, and postpartum family planning. Because constantly different methods of family planning, mental health services, and GVB services are meeting the needs of the community's people, the family planning methods are not only meeting the needs of the facility; they are also meeting the needs of the community.

**Q11: What would it look like if they were to meet the community’s needs fully?**

**Answer:** As we provide the services at the facility, we do not charge any fee for providing the services. And the community health workers we have are always going to the field; follow up with the patients there and send them to the facility free of charge through our own transportation. Thus, we are providing proper quality service.

**Q12: What are three main things that need to happen to make the services more affordable, acceptable and fit for purpose?**

**Answer:** As we bring clients from the community to the facility and refer them, we provide our own vehicles, like CNG or ambulances, and the micro. With these vehicles, we bring them, and if we could provide more help or more transport facilities, of course it would be good for the community and for the pregnant mothers.

**Q13: How does the referral system work? What happens when this functions well?**

**Answer:** Our referral communication methods are, first, through our community health worker bringing the pregnant woman to the facility through our transport. If the mother has any complications, we send her to a higher facility. Of course, we call them before sending them to the higher facility, and sending them to the facility is contingent on their answering the phone. The patient is then transported by us to Bimong Tu Simong with a midwife. Even after the referral, we keep in touch with the mother every day through her mobile phone. And after they come back from there, we bring them back to the facility for follow up.

**Q14: How are referral pathways integrated within the health system? What would an ideal referral system look like? (How is the information communicated and shared with all levels of care? How are service users told about the referral system?)**

**Answer:** We have every type of service in our facility; every data and every document is in our facility, and it will be good for us if it can be based on an online system in the future.

**Q15: What data management systems do you have and could these be strengthened?**

**Answer:** The service that midwives provide to every mother in this delivery service center and the equipment we have are supported by our government. And UNFPA fully supports us. If this support comes in a more proper way, then maybe we can ensure the services for more mothers.

**Q16: How are supplies and equipment for providing high quality services at MLCs ensured? What does the supply system look like when supplies and resources are fully provided?**

**Answer:** Here, we have a midwife supervisor to supervise the midwives for the services they provide. We have a midwife supervisor and a specific clinical mentor to monitor the midwives who are doctors, along with a midwife coordinator and a medical officer to supervise the mentoring. We have medical coordinators to supervise the medical officers. We ensure the quality of each care we provide in this way.

**Q17: Describe the ideal staffing levels for MLCs (workforce numbers and cadres)?  What needs to happen to make this a reality?**

**Answer:** There are supervisors among the midwives who mentor or supervise them. And the report of mentoring or supervision is reported by the midwife supervisor, midwife coordinator, or medical officer.

**Q18: Who is in charge of the MLCs? (If midwives or nurse-midwives, ask for more information about which cadres of midwife or nurse-midwife and how they are different from any other type of midwife that exists in the country) What does being “in charge” look like? (Are the in-charges autonomous, or do they report to a higher authority?)**

**Answer:** Yes, in terms of manpower and infrastructure, it is clear that in rural areas, the HR manpower is relatively low. If manpower and the number of midwives are increased a little more, we may be able to serve more pregnant mothers. In terms of infrastructure, it is often seen that there is a small facility; we provide many types of services in three or four rooms. The infrastructure to ensure that service is either government infrastructure or our own. If the centers are made bigger by UNFPA, then maybe we can make the service more qualitative.

**Q19: What are three things you would change in terms of workforce and infrastructure?**

**Answer:** Since midwives are present in every facility, if there is a doctor in each one to provide some guidance to the midwives, and in the event of a patient referral, if the partner in the facility has the necessary assistance to see any patient at night, a higher quality service may really be provided.

**Q20: What factors facilitate effective coordination and partnership between MLCs and other types of health facility? Please give examples. How could this be improved?**

**Answer:** In order to provide every pregnant mother or client a higher-quality or more competent service, midwives may regularly train, refresh, or orient them to make them more stable. Midwives-led delivery centers must have a comprehensive understanding of the services they provide.

**Q21: What factors facilitate effective coordination and partnership between midwives in MLCs and other health professionals involved in maternal and new-born health care? Please give examples. How could this be improved?**

**Answer:** Every facility where midwives serve patients through evidence-based care must have a standard operating procedure book. Midwives follow that book, and that's how they ensure service to every mother. Furthermore, every facility has guidelines.

**Q22: What competencies should providers have to work within an MLC? What needs to happen to make this a reality?**

**Answer:** Midwife-led delivery centers provide all types of services, such as antenatal care, normal or general delivery, postnatal care, planning services, mental health services, GVB services, CMS services, or any service. And to improve it even more, if we ensure equipment or medicine according to time, then maybe we can provide better service.

**Q23: In what ways are the services provided within MLCs evidence-based? (What guidelines or standard operating procedures exist (if any), and do all providers follow the guidelines?) How do you know?**

**Answer:** Evidence-based services provided regularly by midwives are regularly followed up. For example, we have an online Google sheet or a management system that tracks how much a midwife is delivering regularly, whether evidence-based services are being provided, and whether they are providing services according to the guidelines or SOP. We maintain a report using appropriate documentation and data management.

**Q24: What does high quality care in MLCs look like? What factors facilitate high quality care in MLCs?**

**Answer:** Although midwives read everything in their student life in midwifery, they must constantly do some work for capacity building, such as orientation or training, refresher training about each topic or service, and of course, it will be better if it is an onsite orientation.

**Q25: How is the quality of care assessed and evaluated?  (probe for quality of data and data management systems)**

**Answer:** The maternal mortality rate in Bangladesh has steadily declined ever since midwives began to provide care.

**Q26: If you were planning to improve the quality and efficiency of MLC services, what would be the three main things you would** **do?**

**Answer:** Midwives are constantly doing all kinds of checkups, postnatal care, and deliveries. In that case, midwives are providing some practice- or evidence-based services in some cases. Due to which the quality of service is increasing. For instance, if a mother is bleeding or goes into shock, as they use Non-Pneumatic Anti-Shock Garment (NASG), maybe many of us did not know that. Through midwives, the maternal mortality rate of that mother is decreasing without going into shock.

**Q27: What 3 aspects of MLCs in this country? (Probe: Is there something innovative and unique that is provided as part of MLC services, and other facilities may not deliver that? (Apart from regular services you are providing) Do MLCs serve communities who might otherwise not be able to access care?)**

**Answer:** Missing

**Q28: What modern approaches or technology are used as part of MLC services? Please give examples of how these have been useful/valuable.**

**Answer:** Missing
