# Supplementary material for: Midwife-led birthing centre in the humanitarian setup: An experience from the Rohingya camp, Bangladesh
Source: PLOS Glob Public Health. 2024 Dec 10;4(12):e0004033. doi: 10.1371/journal.pgph.0004033 (PMC11630605; doi:10.1371/journal.pgph.0004033)
Supplement: S2 Data — (DOCX) [file pgph.0004033.s007.docx]

***FGD: Midwives, Rohingya Camp-4, Cox’sbazar***

**Introductory questions:**

**Q1: Explore the respondent’s role in the MLC, their gender, how long they have worked there, was it their own choice to work there or were they just deployed there?**

**Answer:**

I am Asma Akhter. I am working here as a midwife supervisor. I have been here for 2 years and 2 months. And here I am appointed according to my own choice, no one forced me.

I am Kajli Akhter, and I am in this service center as a midwife supervisor. I have been working for one year and eight months. And here I am appointed.

Afifah Sultana, I have been a midwife for over a year.

I am Khadija, working as a midwife. I have been working here for about two years and two months. No one forced me here; I came here by myself.

I am Bristy, I have been here for seven months as a midwife. I've chosen to be here.

I am working as a midwife at this center. I have been working here since last year, and here I am appointed from office.

I am Rima, here working as a midwife. I have been working here since last year. I came here by my own choice.

I am working here as a midwife. I wasn't assigned here; I came on my own accord.

I am Shakila Arefin, and I am working as a midwife supervisor in this service center. And here I am since June 2021. And of course it's a center of my choice, and I love working here.

**Q2: Describe your colleagues at the MLC - who else works with you, how do their roles and responsibilities compare to yours, who is in charge (and what does “being in charge” look like)?**

**Answer:**

I am Asma Akhter. I am working here, and I have midwives as colleagues, along with a community paramedic. And there are paramedic mentors, 2 registrars, outreach workers, and CSWs. Dr. Fatema is in charge of everyone here. Her presence at the facility is really important.

I am Kajli, and I have midwife, paramedic, community paramedic, registrar, CSW, outreach worker, supervisor, paramedic mentor, and in-charge doctor working with me. Having a doctor is very important. Because when we refer to an emergency situation, then surely the doctor helps us a lot.

I am Afifah Sultana, and I have many colleague midwives working with me. Our center has an in-charge doctor, community paramedic, paramedic, guard, cleaner, 2 registrars, and CSWs. It is very important to have a doctor, because with any of our critical patients, she actually helps us a lot in terms of management and referral.

I am Khadija, there are all like others in my camp such as midwife, CSW, paramedic, community paramedic, everyone. But there is no doctor as in charge here. There is a midwife supervisor; she helps us with all the work, and she is very necessary for us.

My name is Bristy, and I work in a camp with a midwife supervisor, CSW, and paramedic. There is no doctor, but we have a supervisor. But she helps us a lot with any work at any time. But there was a need to be in charge.

I am Rimi Akhter, and I have many colleagues in this service center. They are like my family. There are midwives, community paramedics, 2 registrars, family planning mentors, guards, cleaners, and a doctor is in charge of this service center. Midwives play the main role here. If we have to take any decisions or need any suggestions, we can ask for help from the person in charge. It is important to have someone in charge of this.

My name is Rima, and I work alongside paramedics, community paramedics, registrars, outreach workers, lab assistants, and lab technicians. Moreover, there is a guard cleaner. We also have a doctor as in-charge of the center and a midwife supervisor. We, the midwives, work in the facility, and when a patient comes in with a critical situation, we discuss with them what we cannot handle and resolve the issue. It is very important to have someone in charge of this.

My colleagues in this service center are midwives. I am midwife Momina. There are many of my colleagues in this service center, such as midwives, supervisors, outreach workers, lab technicians, paramedics, community paramedics, guards, cleaners, and the in-charge doctor and midwife supervisor. In fact, it is necessary to have midwives, supervisors, and doctors, and we can take their help in any critical situation if we cannot handle it or do not understand it.

I am Shakila Arefin, midwife supervisor at this service center. Apart from the midwife supervisor, I have many colleagues working with me in this service center. If I may say so, there are doctors, paramedics, community paramedics, registrars, lab technicians, outreach workers, health educators, and CSWs. I believe they all contribute through their respective responsibilities. And if I compare my role at work with theirs, I think they try their best to provide a good service. I try to do my best as a midwife supervisor. They also provide paramedic and family planning services if I assume their responsibility. And if I think about lab technicians, then they look at the investigation aspect. As the camp in-charge, I am the midwife supervisor, and the doctor who is with me also plays a role. I think a facility needs a person to be in charge of it. And someone needs to take charge of that facility so that if any of us have a problem—as many of our colleagues work together here—we can share it with the person in charge and we can resolve the issue by discussing it.

***Key questions for focus group discussion:***

**Q3: How does the government support MLCs? How do other organisations (e.g. NGOs) support MLCs?**

**Answer:**

I am Asma Akhter. I think the government supports 100% midwife-led delivery centers. And like the ones in the Rohingya contest, there are some NGOs that work only on maternal and child care.

I am Kajli Akhtar, and Asma Apa is saying the same thing to me.

Afifa Sultana, my answer is as Apu says.

I am Khadija, as Asma is saying, and this is how I am talking.

I am Bristy. Government midwife managed services are, by recruiting through the government, supported by the government with separate labor, ANC, and PNC rooms to work well in health care in any part of Bangladesh. If there is a problem, then the NGOs in the Rohingya context provide good service to the mothers separately. Midwives provide beautiful services for healthy mothers and healthy babies. Then, among many services, they give medicine.

I am Rimi Akhtar, and I agree with Asma Apu and Bristhy Apu.

I am Rima. I agree with Asma and Bristy.

I am midwife Momina. I agree with Asma Apu and Bristy Apu.

I am Shakila Arefin. I agree with Asma Apu and Brishty Apu. I would also like to add that if our Bangladesh government had not supported the centers led by midwives, we would not have been able to work so well since 2012 until today. And with that, if I talk about various NGOs, and especially if I talk about my NGO, RTMI UNFPA, we work with some government facilities in various locations. I think this has been possible only with the efforts of the government and NGOs.

**Q4: How do you engage or involve the community? (How do you get buy-in from community?)**

**Answer:**

I am Asma Akhter. As I work in the Rohingya context, the involvement of service centers here is at the community level. This is done by the entire CSW workers; they resolve through the language variants they have. They work in the field at the community level and inform us.

I am Kajli. This is how we relate to what Asma is saying.

I am Afifa Sultana. We also work like Asma Apu said.

I am Khadija. Asma says this is how we work.

I am Bristy. We also work the way Asma Apu works.

I am Rimi Akhter. I agree with what Asma Apu. Then they bring the information from the community and have a meeting with us, where we discuss and try to solve the gap that exists, or if we can't, we give the information to the supervisor or the person in charge.

I am Rima. I agree with what Asma and Rimi are saying.

I am midwife Momina. We work with the community to collect information from CSWs, outreach workers, and the Majhis and Imams here.

I am Shakila Arefin. I agree with what Asma Apu and everyone else are saying. And with that, we engage in this service center by communicating with the leaders that we have in our community in addition to our field-level workers and providing them with a variety of information and people in the community.

I am Rima. Sometimes it happens like this: A patient comes here. She talks to us and receives a service after coming. Then, after receiving the service, she took some information from the service worker to the community. She went there and informed the people around her that she had taken benifit from this Apa. I was coming here, and you also go for this benefit. In this way, communication is also done through the community.

Furthermore, we have separate sessions with mothers who come for checkups or mothers who stay on admission. We inform them about the community. We tell them in detail how we provide services. This is how we communicate with them.

**Q5: What does an ideal relationship between an MLC midwife and her client look like?**

**Answer:**

I am Asma Akhter. A client must have a friendly relationship with a midwife. And to achieve this, a midwife must maintain the confidentiality of the patient, and if she talks to him in a friendly manner, the patient will be satisfied and share everything with her.

I am Kajli. Respectful care can be provided between a midwife and a client through a good relationship. Even in that case, the patient can believe me and tell me everything. If I can maintain her confidentiality, I must have a good relationship with her, and I always have to make a client feel like she is a member of my family. I have to serve every client the same way I would serve my family.

Afifa Sultana. A client's relationship with a midwife, like that of a mother and child, must be cordial. For that, a midwife must be confidential in the care she gives her. She must be helpful.

I am Khadija. There must be respectful care between a midwife and client. Then privacy and confidentiality have to be maintained; if we maintain these, then a mother will tell me everything, like what is wrong with her. They frequently come to us with many things they are unable to say. They have family problems that they cannot share with us. If we want to understand people's mental problems, we must treat them with respect. As with them, mothers often do not want to say much in front of us. More counseling should be done with them; counseling should be done in such a way that the people of her house, her sister, or her daughter can say that they can share everything with me.

I am Bristy. The relationship between a midwife and a client needs to be very good. By counseling a client, we will reassure her that she can trust us and feel like a member of the family. And they can tell us about their problems in their own way, and we will try to solve their problems as best we can.

I am Rimi Akhter. I agree with all of Apu. A client-patient relationship should be cordial and trusting.

I am Lima. I agree with the others.

I am midwife Momina Begum. The relationship between a client and a midwife must be friendly, and I will provide a service that is respectful and of high quality. And I will counsel the client in such a way that she trusts me properly and I can earn her trust.

I am Shakila Arefin. A midwife and client must have a friendly and trusting relationship so that the client can trust the midwife. And through this, I think the midwife can provide a quality, respectful maternity service.

**Q6: If you were a pregnant woman, what factors would make you trust the MLC services? (Why?)**

**Answer:**

I am Asma Akhter. A pregnant woman will come to my center, and I will ensure her confidentiality and privacy. I will try to gain her trust while ensuring respectful maternity care. That's why she can trust me.

I am Kajli Akter. When I provide a pregnant mother with respectful maternity care and quality care, she must come to me when my respectful maternity care includes confidentiality and personality so that I can trust her.

I am Asma. Apu and I agree with Apu.

I am Khadija. I agree with Asma and Kajli.

I am Bristy. When a pregnant mother first comes to our service center, we will give her respectful and quality care. Then we will reassure the mother, "When you come here for a checkup, you will have a healthy baby and a healthy mother."

I am Rimi Akhter. Pregnant mothers will come to us only when we inform them about their health and when we can inform them about the services we provide and the sessions. Trusting in us then they will definitely come to us.

I am Rima. A pregnant woman will come to our center with confidence based on some of our things, which are basically our behaviors: how we will receive them, how we will talk to them, how we will provide the service, how we will serve them better, etc. If we provide services that are entirely based on their needs and opinions, they will trust us, come to the center, and bring others with them.

I am midwife Momina Begum. When a pregnant mother comes to me for her first checkup, if I treat her well, if I respect her, if I provide my services well, if I receive her well, and if I tell her what services I provide, then that mother will come.

I am Shakila Arefin. I agree with everything they are saying. As a midwife, if I behave well with a pregnant mother, maintain her privacy and confidentiality, and provide good communication and good service to her, then I think she will trust me and come to our center. And she must accept the services we provide.

**Q7: How do you address the specific needs of the community? (Are there any barriers or enablers?) What would you do to make the services more acceptable by the community?**

**Answer:**

I am Asma Akhter. Not all of the specific needs of the community can be met by one facility. We try to fill as many gaps as possible; for example, as ultrasonography is not available at our facility, we refer patients to other facilities or hospitals. In that case, we have this one obstacle. And when other mothers come here with other complications, usually in the PNC center only normal deliveries are done, so when we are referring them to other institutions or secondary hospitals for CS, the attendance of those mothers or that patient does not want to be referred. In that case, we face many problems. But when they go to a more severe stage, they are referred. They are being referred, but we have to do a lot of counseling; that's it.

I am Kajli. What Asma is saying is right. We face many obstacles with this. When we offer long-term family planning methods to those individuals, they refuse. Then they say different things, like that my husband did not give me permission, etc. We face many such obstacles. But then, to avoid this problem, we often arrange a meeting with the majhi or imam. They are counseled on how we can adopt a long-term method for family planning. If we help them comprehend, it is evident that, at the level of the community, they are aware of this. They then disseminate this information at the community level. This makes things a bit easier for us.

I agree with the Apu.

I am Khadija. First of all, the facility I am in and the community I am in, there my position is related to health. There is no delivery center. Then, when my ANC mothers come, their demand is that the delivery take place in this center of mine. Because we often calculate the pregnant mother's EDD, we refer them to D5 as in our other sectors. Then they do not go there. They say I have to stay here; why should I go away? I don't have this delivery center. Even then, I do a lot of counseling for them. Then, with the transportation system that I have there, the vehicle does not go inside the camp. Many times a pregnant mother has to be carried on the road on the shoulder, then she has to be taken from the road to the car because the ambulance cannot enter. This is the problem. Then the camp is a little inside, which makes it very difficult for mothers to come. Then they have the same problem with family planning. They don't want to take a long term method. They need husbands. Then we have sessions with Majhi imams. After taking the session, we talk, and then they tell us, "Okay, I will do it." Another issue is that if a long-term method is used, they return after 2 or 3 months and ask to remove it. For example, they have a problem in the community; one says that family planning is a problem if the mothers do not open it for a long time after they leave. For those, they come back to us after two or three months. Then those have to be opened. After being removed, they become pregnant again. Their children become too many, there are many problems. That's the problem in my camp. I would prefer a health post-delivery center.

I am Bristy, I agree with the Apu.

I am Rimi Akhter. I agree with everyone else. I want to add something else. How we fulfill certain needs in the community: In the community, we are providing service, but they are asking us for something in return for service, what can we get when we come here. For example, in ANC, as we give some medicine to ANC mothers, they come to us for this medicine. Then she will come on the next visit, and medicine will be given again on the next visit. They come with this hope. We can't bring them if we don't give them medicine. As with NVD, we will do NVD; that's how we assure them of that.

If you do NVD here, your child will be gifted something. Otherwise, they will not come to the center and make the delivery. Then the case of PNC is the same; when we bring mothers for a PNC checkup, we also give them some medicine or a physical examination during the checkup. We try to meet the specific needs of the community by providing these services to them. And since we have our meeting in the admissions corner, we have a session every week for admitted mothers. We try to keep their family members, such as heads of families or mothers-in-law, here. We inform them about their health. And we explain to them the advantages and disadvantages of delivery to the facility. We sit here and have a session for them.

I am Rima. I agree with the others.

I am midwife Momina Begum. I also agree with others.

I am Shakila Arefin. I agree with everyone. I want to add something to it. If we look at meeting their needs at the community level, if we take from the ANC, we have some shuttle services that go to the community and bring mothers from the community to our center. And if it is in ANC, we take delivery, and then we have our service called Matri Seba. For any maternal-obstetric case, we bring mothers from the community, and we also refer mothers through this service. It helps us a lot in meeting their many needs. And everyone else who mentioned that we aim to meet the requirements of the community by including mothers, inviting mothers-in-law to sessions, and providing mothers with various types of health education.

**Q8: What is the main payment method for service users? Which women can afford MLC services? Does the government or other organisations arrange any financial support for the service users? (explore in detail)**

**Answer:**

I am Shakila Arefin. Beneficiaries who come to our center for services do not have to pay any fees. At our centers, we bring them here from the community through our shuttle service, ERTS, and our CNW volunteers help bring them to our centers. And after they come to the center, we provide them with the service completely free. As we work with NGO organizations, RTMI is fully funded by the UNFPA fund, from which we cover the cost of services for them. Recently, we have come to know that we refer many mothers to Cox's Bazar Sadar Hospital or Ukhia USC, and since they are refugees, even if they go there, the service is provided free of charge. But it has recently been reported that the RHU has been told that the free emergency and matching services that it used to provide will not be provided or that it will face much difficulty in providing them due to reduced funding. If some organization like this works again and helps them, then I think it will be good; they will get good service. And since they don't want to refer anyway, if they are not given such benefits, it will be seen that they will not want to refer further. And they are not really aware that way, in that case they don't understand how much they will lose if they don't go to refer or get better service.

Bangladesh's government must play a role here. It would be better if the government and NGOs came together to help.

I am midwife Momina Begum. I agree with Shakila.

I am Rima. I agree with Shakila.

I am Rimi Akhter. I'll urge cooperation between the government and other NGOs, especially with regard to the Rohingyas, that's all.

I am Bristy. I agree with others.

I am Khadija. Actually, I agree with others. And the money is not really needed. But if other NGOs work, then they get some help.

I am Afifa Sultana. I agree with others. Mothers don't need any money to come to our center; we provide them with services free of charge.

I am Kajli Akter. When the Rohingyas also come to us for services, they do not have to pay any money. We carry it in the RTMI UNFPA fund. It would be good if the government and other organizations helped them in this.

I am Asma Akhter. I agree with everyone.

**Q9: How do you ensure that the MLC has all the supplies, equipment and resources it needs to provide high quality services?**

**Answer:**

I am Shakila Arefin. In our service center, we work in the PHCC (Primary Health Care Center, A and B1 levels). Our center provides the following services: ANC, normal vaginal delivery, family planning, PNC, and others. And through it, it can provide a quality service. But since we work at PHCC, we must refer to the Simong facility. And many times, it is seen that a mother has PPH at delivery. We may be able to manage PPH in a PHCC, but if she needs blood transfusions at a later stage or if a mother needs a caesarean section, we have to refer her to a Simong facility, and we face a lot of problems here; for example, mothers don't want to go, and we have many other problems. In an emergency situation where Simong facility is far away, a risk is left for the mother. And the thing about ultrasound is that we have to send mothers to Ukhiya UHC and other places for ultrasound. In this case, if all these facilities were in our PHCC, we could provide better services, and if a mother came to us, we could fulfill all her needs.

I am midwife Momina Begum. I agree with Apu. But I would like to add something else. It would be great if we had a baby warmer or sucker machine here.

I am Rima. I agree with others.

I am Rimi Akhter. I agree with them. But as Apu said, a baby warmer can't capture the baby's temperature. Therefore, if the baby's condition is a little worse or if it has hypothermia, it has to be referred for such small equipment. If such equipment were available to us, our problems would be reduced. And mothers come to us since the hospital is near their home. Here, they want to get all the services. They don't want to go elsewhere easily. For this reason, having those all here would be beneficial for us.

I am Bristy. I agree with Apus.

I am Khadija. I actually agree with Apus. But if there is a delivery center in the center that I have, then it is better, and mothers do not want to go out to deliver after coming for a checkup at the delivery center. They want to deliver a baby at my camp. But I don't have a delivery center here, so delivery is not being done. I find it more difficult to refer mothers. I frequently overhear them telling me they are having labor pains on the way home. After leaving to visit the facility, I hear that the delivery has been done. For this, it is better if I have a delivery center here.

I am Afifa Sultana. I agree with the others.

Kajli Akter. I meant all that was stated.

**Q10: What kind of information do you record about MLC performance? (probe for central data management system and if they comply with data collection requirements)**

**Answer:**

I am Asma Akhter. When an ANC mother visits our service center, her complete medical history, as well as her obstetric history, are entered into the total history database.

Kajli Akter. When an ANC mother comes to us, we make a complete entry, from her name and age to her husband's name and address, and we have a register. If an ANC registry mother is also an ANC patient mother, we keep the ANC registry and have EDD tracking. When we midwives check up the mother after ANC registration, after the checkup there is a matter of calculating the mother's EDD. There are numerous others. Well, ok, I'm giving the next one.

Assalamualaikum. When an ANC mother comes to me, we maintain the ANC register; if it is NVD, we maintain the NVD register; if the PNC mother comes, we maintain the PNC register.

I am Khadija. I agree with you. First, ANC mothers are actually registered in the ANC register, then ANC, PNC, family planning mothers who come, they have separate registers; we enter them in the register.

I am Bristy. I agree with others.

I am Rimi Akhter. How we collect data at our service center: When an ANC mother comes to us, we take her obstetric history, LMP EDD, and all her history and manage a blood donor for her. Also, the register that we keep. How many weeks is she due and when will her EDD be; we maintain such ANC registers, and if it is NVD, we maintain the number of registers we have for NVD. There they have general examination and obstetrics, then far abdominal far general and we have the data to enter. We maintain them all. Even at PNC, we maintain several types of registers. This is how we collect their information.

I am Rima. When a mother comes to our center for an ANC checkup, first we maintain our ANC register with her history, her name, her age, her husband's name, Majhi, all the history; and she has a checkup book, in which we make entries. When they come in for ANC checkups every month, we maintain the ANC register book. When a mother comes for delivery, we maintain the delivery register after delivery. If the mother has any high-risk factors, we keep a record of those in the high-risk register. We maintain a sick register if the mother comes up with an illness without a checkup. Then, when we do a PNC checkup on the mother, we maintain the separate PNC register. Then, when we refer a mother, we keep the referrer's referral record. Also, if a mother first comes to our center for delivery, we enter her in the admissions register. Then comes the service: If we give them medicine or something else, we record it in the medicine register. Then the gift we give them can be a mama kit, a hygiene kit, or a minister hygiene kit; we maintain a separate register for these. And in all the registers, we enter their name, their husband's name, their shade number, and their block number.

I am midwife Momina Begum. I agree with Rima.

I am Shakila Arefin. I agree with what everyone has said so far. As it is an antenatal care center, here we provide all types of ANC, PNC, and NVD services. They have said a lot about the ANC. In between, we maintain another register, the follow-up register, in which we keep all the data about the mother. Later, we use the follow-up register to check whether the mother is coming to the center properly. And by looking at that follow-up register, we try to follow up on whether the mother can take all the visits or not. And in delivery, we maintain a register called Evidence-Based Practice, which starts with a mother's labor pains.

**Q11: How do you manage emergency/ complicated cases? (Probe: Where do you refer the client? What kind of agreement with other health facilities do you have? How do they get there?) What does it look like when the referral system works well?**

**Answer:**

I am Asma Akhter. Our critical patients who arrive with complications are referred to the nearby Past Hopehill Hospital; obstetric cases are also referred there. Then for the baby, we refer to MSF Hospital Kutupalong; if it is gynecologically related, we refer to Cox's Bazar. Our emergency referral system is ERTS, and our transport referral system has an ambulance service. By doing this, our midwife accompanies the patient, and when the referral arrives at the facility, the midwife carries the total data documentation.

I will add to what Asma said. That is, the hotline number of the center to which we refer is given in all our centers. We always contact the hotline number before we refer someone. Tell them that we have a critical patient who is going to their facility, and they should take care of that patient. If we can refer a patient in a timely manner, both mother and baby will be saved. And if we contact the referral center in advance, then they will be prepared in the same way. Then it will be very good; both mother and child will be healthy.

When we refer a patient, we always call the center first. We, the midwives, go there after calling them. We document what the mother is receiving when we visit the facility; we mention it including the time. After going to that hospital, we handed everything over to the midwives. We keep that mother's phone number even after she has been handed over, and we keep in touch with her to investigate her condition. Even after she returns home, we continue to focus on her outcome.

I am Khadija. Mothers who are referred to my camp first go to my RTMI D-5. Later midwives refer from there. And when I refer, I send a CHW with that patient through my ERTC, which is the vehicle. Because I don't have a midwife here, I work here alone. This is why the midwife does not go. But when the patient goes to another center of RTMI, if referral is required from there, the midwives go along.

I am Bristy. I agree with others. When we first refer a patient for a complication, we tell the hotline number that the patient should not have any problems. If there is CS, we go to Madukhali, and if there is any heart problem, they don't want to take it. Then we refer to Cox's Bazar.

I am Rimi Akhter. I agree with you. I will only talk about how well the referral system works. They are prepared in advance to receive our patients by calling the hotline number. We go to the facility, hand over the patient, and take their phone number. For example, we refer our 'tear-clean' patients very quickly to primary management. After going there, they do a c-section or a normal delivery quickly. With this system, we can save lives; otherwise, we wouldn't be able to. Thus, this referral system brings very good results.

I am Rima. I agree with you.

I am midwife Momina Begum. I agree with you.

I am Shakila Arefin. I agree with everyone. Here's how we manage urgent or critical patients: As our center is a PHCC, we provide as much emergency management as we can at a PHCC. And we refer patients to the Simong facility. Mainly, we refer to Hopefill Hospital, and at the time of referral, our ERTS ambulance is accompanied by a midwife. And we have a box called the referral emergency box, which we carry with the ambulance. And here we have some medicine and some instruments according to the cases that we usually face as a maternity service center. During our referral system, if we ever have a problem with an ambulance on the road, we can manage that mother immediately. For this, of course, we take the midwife and the box with us. And since the midwife is present when the patient is handed over after arriving there, she can give the patient's history very well. Of course she takes the documents, and we already contacted the referring center to know what kind of patient we were referring and what it would take; of course we shared the patient's history with them. And we tell them in advance, because of which the mother gets the service according to her needs.

**Q12: What are the best things/moments for you as a provider working in MLC? (What do you enjoy the most?) How do you feel about working within MLC? What would you like to change in future to make the situation better for you? (probe for burnout, supports and workload)**

**Answer:**

I am Asma Akhter. Every day, we feel good here. Because the feeling is definitely good when there is a healthy mother and a healthy baby after delivery. Especially in the case of critical babies like those with birth asphysia, if the baby cries beautifully after receiving HBB, that feeling is special to everyone. A midwife thinks this is best. Another thing I'd like to change in the future is to have one or two rooms like the breast feeding corner we have in the PNC room, but our space is so small that if we have a lot of patients breastfeeding there, it becomes crowded. A separate room would be preferable.

I am Kajli. My favorite moments here as a service provider are when I manage critical deliveries like breech deliveries and solder dystasia cases. I like it a lot. And another thing is that my center does not need any changes; everything is fine, Alhamdulillah.

Afifa Sultana. My favorite thing as a midwife is when I hand the baby to the mother after delivery and the mother takes her baby with a smile. And if our centers have a breastfeeding corner, then it will be better.

I am Khadija. It's good when a mother holds her baby after delivery, but if I change camps in the future, it's better for me if it is made a delivery center.

I am Bristy. My center has a delivery room, but ANC and PNC are all in one place. Had it been separate, the mother's privacy would have been maintained, which would be good.

I am Rimi Akhter. I agree with Asma.

I am Rima. I agree with others. But it's nice as a midwife when we deliver a mother and the baby, and the mother is healthy; when we put the baby on the mother's skin and she's breastfeeding, the mother smiles; and when the baby is healthy and beautifully breastfed, I really feel happy. I am proud to be a midwife. And I think we are competent midwives, but it would be better for all of us if we could be made more competent through adequate training.

I am midwife Momina Begum. I agree with Rima. However, my favorite moment as a midwife is when a delivery takes place and the baby cries as it happens.

I am Shakila Arefin. As a midwife, my job is to keep mothers and babies healthy and reduce the maternal mortality rate in our country. Of course, we work for this. And then it is good to think that those moments are much better if there is an emergency situation or if the mother is in a life-threatening condition and we can help her survive. I feel that I am doing very well, and I am very happy to work in this service center. And it is clear from the context that the midwife is somewhat unfamiliar in any service competition. If we can all make midwifery better known, it seems that our work environment will improve.

**Q13: What competencies do providers need to work in an MLC? Do you think all health care providers in this MLC have all the required competencies? If not what should be done to improve it?**

**Answer:**

I am Asma Akhter. I think all the staff at the service center is experienced and has skills. Still, capacity building needs to be done by training everyone to increase their skills.
 
I am Kajli. What Asma told us is that by building up our capacity through various trainings, we will be able to provide better services.

Afifa Sultana. A midwife must have the skills to manage a PPH patient with a critical condition. For example, if a baby is born in the birth space, the midwives must know how to keep the baby alive and how to make the baby cry. It is important to know what the baby needs after a breech delivery, what can happen after the baby is born, and what is needed. I think every midwife is competent to use the instruments available in the centers, and training is needed to improve their skills.

I am Khadija. All of us midwives here are skilled. But sometimes twins are delivered, the baby is upside down, and sometimes we have to do forceps delivery, in which case we midwives get nervous. As many times as there is no doctor at night, we handle it by ourselves. Then it becomes a little difficult for us. For this, it is better if we can improve our skills through training.

I am Bristy. I agree with Khadija.

I am Rimi Akhter. Our midwives are all skilled. But still, we face many kinds of problems. For example, many babies are in the breech position during delivery, and severe preeclampsia is present. Many a time, we ourselves get very nervous looking at the patient's condition. Our doctors are not always available, for instance, on the night shift. It is very good to have a doctor on the night shift. And midwives must have critical thinking and emergency case management capabilities. I need the necessary skills for health care services in this service center. There is no doctor on the night shift, so it is better if there is a doctor available.

I am Rima. I agree with the others.

I am midwife Momina Begum. We are all experts here, but I think ... I agree with the Apus.

I am Shakila Arefin. We, the midwives, are working in our service center. Since it is a midwife-led care center, midwives usually provide a variety of services. And they have sufficient expertise in the services they provide. Among them are some urgent conditions of the mother, in which the midwives are skilled; for example, PPH, severe preeclampsia, birth eclampsia, prolonged labor, obstetric labor, low birth weight, and how to manage the baby—these things must be done efficiently by midwives. Many times it is seen that they manage breech delivery and shoulder dystocia very efficiently, but in this case we have to refer to prolonged delivery. In that case, vacuum delivery and forceps delivery can be done by midwives according to the guidelines. It would be preferable if they provided us with more training.

**Q14: How do you ensure that the care that you provide is evidence-based? What main guidelines and standards do you use? Are those guidelines helpful? What else needs to be done?**

**Answer:**

I am Asma Akhter. I'm a registered midwife, and I have passed the BNMC exam and received a certificate; that is my evidence base. And in my facility, we have doctor guidelines as guidelines, as well as SOPs that we follow. Also, if guidelines-related books are provided in our center, then maybe it will be good for every midwife.

I am Kajli. I am a registered midwife of the Bangladesh Midwife Council. My center has different types of SOPs. I work according to those and if there are charts, banners, or festoons as aids, I can use them to counsel mothers daily and take sessions of various types.

I am Afifa Sultana, a registered midwife. Of course, we work according to the WHO guidelines that are there.

I am Khadija, a registered midwife. I got certificate from BNMC. We work according to WHO protocols. And if we are given some kind of book or a book according to the guidance of the WHO, then it is better for us.

I am Bristy. I am BNMC registered. According to what we have learned in 3 years of college life, we document every pregnant mother who comes to us. We then check them up accordingly. And we work in accordance with WHO guidelines.

I am Rimi Akhter. The services we provide are definitely evidence-based. We are certified midwives. If we need to prove it, we have certifications, documentation, and everything else that we can show immediately. We use the WHO guidelines as a guide. Each center has SOPs, which we follow. This guide helps us in many ways. If we forget any guide lines, we can fill them up again by following this guide.

I am Rima. I think the service we provide is evidence-based. Because we are coming here after studying midwifery for 3 years, and we are coming here as registered midwives from the BNMC. We use guidelines; there are WHO guidelines, national guidelines, and we follow those. And our center has SOP. We follow those books and work according to those, and we work based on what we learned.

I am midwife Momina Begum. Of course, the services we provide are evidence-based, as we have completed a 3-year midwifery course and are certified by the BNMC. We work as per national guidelines and SOPs.

I am Shakila Arefin. As a midwife, I am committed to providing evidence-based services. I am a registered midwife, and I provide an evidence-based service. And as proof of this, we must follow various guidelines. National guidelines, WHO guidelines—following these guidelines, we provide evidence-based services, which are definitely evidence-based. And I think the specific guidelines that we use are: if we have an update system at our service center, as medical science is updated daily, we need to know updated topics; if they can be confirmed, then I think it is good.

**Q15: If you were planning to improve the quality and efficiency of MLC services, what would be the three main things you would suggest?**

**Answer:**

I am Asma Akhter. I think gynecological and obstetrical-related trainings are very necessary for our capacity to build rapidly and for the quality of the service we are providing by our midwives.

I am Kajli. I think that to improve our efficiency, we need to provide respectful maternal care and quality care.

I am Afifa Sultana. I agree with Asma.

I am Khadija. I agree with Asma and Kajli.

I am Bristy. I agree with Asma. And the trainings are discussed through our regular sessions, and if anyone has any gaps, those need to be solved one by one through their questions.

I am Rimi Akhter. I agree with them. And I will add one thing. Our facility has a question box. We leave the question that we have questions about in the question box. In that case, when our in-charge midwife supervisor or midwife mentor comes, they explain it to us briefly.

I am Rima. I agree with you.

I am midwife Momina Begum. I also agree with others.

I am Shakila Arefin. As a midwife, if we want to improve midwifery services, what I would say from my side is that we must build up our own capacity. And, in order to strengthen the service, if the government or an NGO can organize a conference or something where many midwives can gather, they will each share how they can expand and improve the service. And if there are international midwives who will be here, as we have had various international midwife mentors in our organization, we are learning a lot from them, especially from Rondi Ma'am, who was also in our organization in her capacity and has been working with these types of international midwives for many years. It is possible to improve the midwifery service we have if we can communicate with them, whether it is through the government or through various NGO organizations. It is better to interact with more advanced countries because it has only been functioning in Bangladesh for a very short period of time.

**Q16: In what ways is the care provided client/women-centred?**

**Answer:**

Of course it is women-friendly, as it serves mothers and babies.

I am Kajli. The services we provide at our center are definitely woman-friendly. Because here we provide ANC, PNC, and NVD services, which are done by women. So I think we provide women-friendly services.

I am Afifa Sultana. The service centers and services we have are definitely woman-friendly.

I am Khadija. The services we provide are definitely women-friendly, as we work with mothers and babies.

I am Bristy. The services we provide are definitely woman-friendly. We provide these services to mothers and children as women because we are also women.

I am Rimi Akhter. The services we provide are definitely women-friendly, as we serve only mothers and babies. And we provide services related to mothers' problems.

I am Rima. I agree with everyone. As midwives who work in midwife-led delivery centers, we must work with mothers and babies. So it is a woman-friendly service center.

I am midwife Momina Begum. The services we provide are woman-friendly.

I am Shakila Arefin. We provide services at our maternity services in a variety of ways, and of course, a midwife is always a midwife with a mother-centered approach. In a variety of ways, a midwife provides services that are mother-focused.

**Q17: Is there something innovative and unique that your MLC provides and that facilities do not? (Apart from regular services you are providing) How does this benefit the clients and/or the health care providers?**

**Answer:**

I am Asma Akhter. Apart from regular services, we also provide some other services like CMR and GBV, which are not regular, but this related service is provided by midwives, and I think it is definitely positive for the survivor.

I am Kajli. Apart from our regular services, we also provide GBV and CMR services. In this case, the service recipient definitely benefited. When a GBV mother comes to us, we refer her to our GBV case worker. Then the GBV caseworker supports her in the same way she does.

I am Afifa Sultana. I agree with you.

I am Khadija. I agree with Asma and Kajli. Because we provide CMR and GBV, which are not always available.

I am Bristy. I agree with the others.

I am Rimi Akhter. Apart from regular services, we also provide MBA, post-abortion care. These services help the mother's health and can save her life. Thus, they get service from us.

I am Rima. Apart from regular services, we provide some services. Those are STIs, RTIs, and reproductive infections, which are very important for a mother.

Apart from regular services, we also provide other services like CMR, GBV STI, RTI, MR Pack, etc.

I am Shakila Arefin. Some services are regularly provided outside of our service center. In our service center, we provide various services in an integrated manner that are of great benefit to the beneficiary. We work with SRS when they come to our center. But apart from this, if they have any problems, our partner organizations provide them with various services. We can provide services according to the needs of the beneficiary in an integrated manner so that we benefit as well as the beneficiary.

**Q18: Is there any** **modern approach or technology that you are using as part of MLC services? How does this benefit the clients and/or the health care providers? What kind of technology or approaches would improve services in future?**

**Answer:**

I am Asma Akhter. Regardless of the services we provide, as a modern technology, we primarily use the fetoscope to listen to the heartbeat. But as a result of modern technology, through the doppler machine in our center, only I can hear through the fitoscope, but through the doppler machine, I, along with a mother, can also hear the heartbeat of her baby.

I agree with Asma.

I am Afifa Sultana. I agree with the others.

I am Khadija. I agree with Asma.

I am Bristy. I agree with Asma. And if the ultrasonogram can be done with advanced means, then we can confirm to a mother what the position of the baby is and whether the delivery will be normal or not.

I agree with them. I will add our thermometer, which we use the digital one. For example, if I show them mercury, they don't understand. As the marking comes in, it helps to convince them that what we are saying is correct.

I am Rima. I agree with all of them.

I am midwife Momina Begum. I also agree with everyone.

I am Shakila Arefin. As part of the midwife-led service, we use modern methods, or if we go into the delivery position, we use the birthing chair, which helps a mother relieve a lot of pain during delivery. And if we deliver the mother using the birthing chair, it is very beneficial for the mother. Also, for various positioning techniques that are used in developed countries, such as birthing balls for pain relief, developed countries use different types of equipment; I think it would be good if such equipment was provided in our facility.

**Q19: How do you facilitate access to MLC services for those who might find it more difficult to access care here, e.g. because they are poor or cannot easily travel? How could you improve this in future?**

**Answer:**

I am Asma Akhter. In our Rohingya context, we provide services to the refugees completely free of cost. We have ERTS Ambulance for everything from medicine to transportation. We also provide shuttle service. Although they are referral systems, we refer through ambulances.

I am Shakila Arefin. The services we provide at our service centers, conducted by midwives, are completely free, and we provide the services to the beneficiary. We have various services for them, such as shuttle service, emergency referral, and transport service, through which they do not need to pay any travel costs. We bring them along with our CSW volunteers so that they can receive these services as needed and are motivated to come to our center and take advantage of the services. That is why we provide these services. If we want to improve it and provide better services, we also have many centers that are in hilly areas, where there are many problems with commuting. In that case, if we can use any alternative method, then mothers can easily reach our center. If we take refugees and camps into context, we have some problems. There is a network issue. There are several issues with the network, for which it takes some trouble to provide the right service at the right time. In that case, if we can solve it and provide better services for them in the future, if we can increase some facilities in the PHCCs we have and provide some advanced equipment and supplies, then they will be willing to accept the service easily. And I think we can give them a good quality service.
