# Supplementary material for: Midwife-led birthing centre in the humanitarian setup: An experience from the Rohingya camp, Bangladesh
Source: PLOS Glob Public Health. 2024 Dec 10;4(12):e0004033. doi: 10.1371/journal.pgph.0004033 (PMC11630605; doi:10.1371/journal.pgph.0004033)
Supplement: S3 Data — (DOCX) [file pgph.0004033.s008.docx]

**Q: Tell me about your most recent birth at (name of MLC).**

**Answer-1**

The name of this birthing center is RTMI, KTBRC Birth Unit.

**Q: When was it? Did you have a son or a daughter?**

**Answer-2**

My delivery was on November 13. This is a baby boy.

**Q: Was it your first birth? If not, where did you give birth before?**

**Answer-3**

This is my second child. The previous child was born here.

**Q: How did you hear about the MLC and why did you choose it?**

**Answer-4**

The counseling provided by the birth unit sisters here is very good. And those who have CSW from RTMI visited our house and told about it. Sister, CSW, and counseling are all excellent. So I chose it.

**Q: What did you like about the MLC?**

**Answer-5**

I like the way of speaking and counseling of the midwives of the birth unit of this hospital very much. They provide service as soon as the patient arrives. It provides services according to the needs of the patient.

**Q: What did you like about the staff of the MLCs? ( feel comfortable to share things or ask questions)**

**Answer-6**

The midwives in the delivery room and the female doctors who stay here are very good at counseling. And if there is any difficulty or need to refer, then they directly provide us the information in advance.

**Q: How did they involve you and your family in decisions about your care?**

**Answer-7**

They were telling my family not to worry after I come here, they will serve us well. In this way, they have cooperated a lot.

**Q: In what ways did the MLC respect your needs? (probe for things like: birth partners, language, respect for cultural traditions that are important to the woman)**

**Answer-8**

I told them I needed the chair for delivery, and they respected my demand. They agreed to my wish. They did not treat me badly. I'm a Rohingya; I'm a refugee, but they didn't disrespect me at all. They saw me as their own sister. I am very proud of their service.

**Q: What or who helped you to pay the costs of accessing care? (probe as appropriate for: user fees, transport costs, food and accommodation for self and family members, medicine costs, equipment costs (e.g. sanitary pads)**

**Answer-9**

It didn't cost me anything. When I started having pain, I called CSW. They brought me in an ambulance. The midwives here delivered me, gave me the medicine, and did everything else that was needed. I didn't have to spend anything.

**Q: Would you recommend the MLC services to other women? If yes or no why?**

**Answer-10**

As I got service here and got well, the treatment is good here, so I will tell others that this place is good, too. They can come here to receive service.

**Q: What are three main things to be changed for better services in future?**

**Answer-11**

It would be better if there was another hospital here where all the services would be available without referring to the emergency situation. Additionally, it would be nice to have an ultrasound machine here. And having a separate room for breast feeding would be convenient.

**Q: Do you think the MLC has all the health workers, materials and equipment it needs to provide high quality childbirth services? What should be done to make it better in future?**

**Answer-12**

It would be better to bring that facility to this hospital instead of referring to a Caesarean or any other emergency for better service in the future. I think it would have been better if those treatments could have been done in this hospital.

**Q: What did the midwives do to make you feel confident that they knew how to do their job well?**

**Answer-13**

I have taken services here since I became pregnant. They have given me proper advice, counseling, and service. In a word, they have taken on all the responsibility of the service until delivery.

**Q: What did the midwives do to make you feel confident in your own ability to give birth safely and care for your baby?**

**Answer-14**

The midwives advised me on how to stay healthy. I went accordingly. And that's why I had a safe delivery; I'm well. So I trust them.

**Q: What documentation and paperwork did they give you when you were discharged from the MLC?**

**Answer-15**

While I was leaving, they gave me the discharge form, some medicine, and instructions on how long and when to take it. In addition, they told me how to take care of the baby and when to come to the hospital with the baby.

**Q: Before you gave birth, what information did the MLC give you about what would happen if there was a complication or emergency that meant you needed to transfer to a hospital?**

**Answer-16**

They advised me to come to the hospital if I have a high fever, leg swelling, neck pain, or slight bleeding during pregnancy.

**Q: Did you or your baby need to be transferred to another facility either during labour or shortly after the birth? Why? Tell me about that experience. How did you feel?**

**Answer-17**

After delivery, they kept me here for 24 hours of observation. They discharged me after giving me medicine. There was no need to transfer anywhere else.

**Q: How did you make the journey from your home to the MLC? What would have made their journey easier for you?**

**Answer-18**

I called the CSWs when my labor started. They brought me here in an ambulance. I had no trouble.

**Q: Would you give birth at MLC again in future, or recommend the MLC to a friend or relative? Why?**

**Answer-19**

Since I have made two consecutive deliveries here, I will come again if needed. And since their service is good, I will ask my friends and relatives to come here too. I will tell them that this hospital is very good and provides good services.

**Q: What are the things that could have been improved further? Please describe three main things you would suggest for improvement.**

**Answer-20**

For the improvement of the birth unit there, everyone, including me, thinks that it would have been better if the caesarean was done here instead of being referred elsewhere. Ultrasound system is also good here. And it would be better if the other services that required going outside were provided here.

**Q: What is it about the MLC that makes it different from other health facilities where women can give birth?**

**Answer-21**

In case of sudden labor pain, pregnant mothers can come here instead of going far; they don't have to spend money, and the midwives are very good. So I think this hospital is safe. It feels good to be able to come here whenever I want without having to go far.

**Q: How did the midwives make you feel respected?**

**Answer-22**

They behaved well, respected my privacy, and showed respect. They spoke to me with great respect.

**Q: How did the midwives encourage you to ask questions and ask for what you needed?**

**Answer-23**

The midwives told me what I needed. They informed me about my EDD. They asked me how I felt and whether my back or neck was hurting. I answered as they asked me.

**Q: How did the midwives encourage you to make your own decisions about your care?**

**Answer-24**

The midwives gave me advice on how to stay physically and mentally well. They said how often I should have a baby. They also told me how to take care of myself and the baby, how to breastfeed, how to bathe, and what to do for a cold. These were appealing to me.
