# Supplementary material for: Midwife-led birthing centre in the humanitarian setup: An experience from the Rohingya camp, Bangladesh
Source: PLOS Glob Public Health. 2024 Dec 10;4(12):e0004033. doi: 10.1371/journal.pgph.0004033 (PMC11630605; doi:10.1371/journal.pgph.0004033)
Supplement: S4 Data — (DOCX) [file pgph.0004033.s009.docx]

**IDI-2: Rehana Khatun, Cox’sbazar**

**Q: Tell me about your most recent birth at (name of MLC).**

**Answer-1**

The name of this hospital is RTMI.

**Q: When was it? Did you have a son or a daughter?**

**Answer-2**

I had a baby this morning. It's a baby boy.

**Q: Was it your first birth? If not, where did you give birth before?**

**Answer-3**

I already had one child before this one. The first child is a girl.

**Q: How did you hear about the MLC and why did you choose it?**

**Answer-4**

Johura Khala is a volunteer. She brought me here. I came here because I will get better after treatment.

**Q: What did you like about the MLC?**

**Answer-5**

I like this hospital very much. I have delivered a baby boy here. I am happy.

**Q: What did you like about the staff of the MLCs? ( feel comfortable to share things or ask questions)**

**Answer-6**

All the staff at this hospital are very good.

**Q: How did they involve you and your family in decisions about your care?**

**Answer-7**

Missing

**Q: In what ways did the MLC respect your needs? (probe for things like: birth partners, language, respect for cultural traditions that are important to the woman)**

**Answer-8**

The staff treated me very well. I have found so much peace here. They took care of me very well.

**Q: What or who helped you to pay the costs of accessing care? (probe as appropriate for: user fees, transport costs, food and accommodation for self and family members, medicine costs, equipment costs (e.g. sanitary pads)**

**Answer-9**

It didn't cost me any money to come here. The hospital gave me medicine and food. In the morning I took what the medicine they gave me.

**Q: Would you recommend the MLC services to other women? If yes or no why?**

**Answer-10**

I will ask people around me to come here for services. Additionally, I will tell them, "I got good service in this hospital. You will do well if you deliver the baby here."

**Q: What are three main things to be changed for better services in future?**

**Answer-11**

The body aches and the head turns during delivery. I could have come much better if there had been a good transportation system. As it is a hilly area, no vehicles can come. Vehicles may arrive if suitable roads are constructed. Otherwise, it is very difficult to come here now.

**Q: Do you think the MLC has all the health workers, materials and equipment it needs to provide high quality childbirth services? What should be done to make it better in future?**

**Answer-12**

Everything is available here to serve me and the baby.

**Q: What did the midwives do to make you feel confident that they knew how to do their job well?**

**Answer-13**

The staff of this hospital treated me very well. They delivered my baby beautifully and took good care of me.

They gave me medicine. They talked to me well.

**Q: What did the midwives do to make you feel confident in your own ability to give birth safely and care for your baby?**

**Answer-14**

Missing

**Q: What documentation and paperwork did they give you when you were discharged from the MLC?**

**Answer-15**

Documents and medications are also given to patients as they leave the hospital. They explain when and how to take the medicine.

**Q: Before you gave birth, what information did the MLC give you about what would happen if there was a complication or emergency that meant you needed to transfer to a hospital?**

**Answer-16**

I was told that my baby will be delivered in this hospital.

**Q: Did you or your baby need to be transferred to another facility either during labour or shortly after the birth? Why? Tell me about that experience. How did you feel?**

**Answer-17**

Me and my baby are fine. So we did not have to transfer to any other hospital.

**Q: How did you make the journey from your home to the MLC? What would have made their journey easier for you?**

**Answer-18**

I came to this hospital on foot from my house. Walking is quite challenging. It would have been preferable if a vehicle had been made available for that. Hills are insurmountable for automobiles. The road has to be repaired for this.

**Q: Would you give birth at MLC again in future, or recommend the MLC to a friend or relative? Why?**

**Answer-19**

If I get pregnant again after this, I will come back to this hospital to deliver my baby.

I will advise any of my relatives to come to this hospital if they want to deliver their baby. I will tell them that they will be better off if they come here.

**Q: What are the things that could have been improved further? Please describe three main things you would suggest for improvement.**

**Answer-20**

Medicines are dispensed from here; the staff are all very good; they treated me well; and the hospital is open 24/7. All is well here. We suffer only from the lack of moving vehicles. Because the road is not good. Vehicles can move if the road is in good condition.

**Q: What is it about the MLC that makes it different from other health facilities where women can give birth?**

**Answer-21**

Here is Johura Khala. She had told me about this hospital. I came here at night. After coming at night, they said that the baby could be delivered here.

**Q: How did the midwives make you feel respected?**

**Answer-22**

The midwives treated me well after I came here.

**Q: How did the midwives encourage you to ask questions and ask for what you needed?**

**Answer-23**

After I came here, the service provider asked me what the problem was. They gave me medicine.

**Q: How did the midwives encourage you to make your own decisions about your care?**

**Answer-24**

My baby is a little underweight. They told me to eat more nutritious food.
