# Supplementary material for: Midwife-led birthing centre in the humanitarian setup: An experience from the Rohingya camp, Bangladesh
Source: PLOS Glob Public Health. 2024 Dec 10;4(12):e0004033. doi: 10.1371/journal.pgph.0004033 (PMC11630605; doi:10.1371/journal.pgph.0004033)
Supplement: S5 Data — (DOCX) [file pgph.0004033.s010.docx]

**Q: Tell me about your most recent birth at (name of MLC).**

**Answer-1**

The name of this hospital is RTMI Hospital.

**Q: When was it? Did you have a son or a daughter?**

**Answer-2**

On November 12, I gave birth in this hospital. I gave birth to a girl.

**Q: Was it your first birth? If not, where did you give birth before?**

**Answer-3**

I have three more children except this one. There are four with this baby. Out of those three, two were born in Burma and one in this hospital.

**Q: How did you hear about the MLC and why did you choose it?**

**Answer-4**

Amina, one of the volunteers, told me about this maternity care center.

**Q: What did you like about the MLC?**

**Answer-5**

 If I come to the hospital, I will feel better; I will get peace from illness. Here is the doctor; they treat people well.

**Q: What did you like about the staff of the MLCs? ( feel comfortable to share things or ask questions)**

**Answer-6**

The midwives of this hospital are good. They have served me well.

**Q: How did they involve you and your family in decisions about your care?**

**Answer-7**

I like coming here. There is no doctor, and it is heated at home. But there are doctors and midwives in this hospital. Coming here is beneficial for me.

**Q: In what ways did the MLC respect your needs? (probe for things like: birth partners, language, respect for cultural traditions that are important to the woman)**

**Answer-8**

The midwives here did not treat me badly. They speak very nicely and serve well.

**Q: What or who helped you to pay the costs of accessing care? (probe as appropriate for: user fees, transport costs, food and accommodation for self and family members, medicine costs, equipment costs (e.g. sanitary pads)**

**Answer-9**

I did not need to spend any money here. Amina Apa had me walk with a rope. Medicines and vitamins have been given to me from this hospital.

**Q: Would you recommend the MLC services to other women? If yes or no why?**

**Answer-10**

I like this hospital very much. So I will ask people around me to come here. They will receive great treatment if they come here.

**Q: What are three main things to be changed for better services in future?**

**Answer-11**

I had a fever and severe pain during labor pain. I couldn't move. After coming here, they gave me medicine, and then I could move. It would be good if more and better medicines were provided.

**Q: Do you think the MLC has all the health workers, materials and equipment it needs to provide high quality childbirth services? What should be done to make it better in future?**

**Answer-12**

Everything is good in this hospital.

**Q: What did the midwives do to make you feel confident that they knew how to do their job well?**

**Answer-13**

The midwives here have taken very good care of me. They talked to me very nicely.

**Q: What did the midwives do to make you feel confident in your own ability to give birth safely and care for your baby?**

**Answer-14**

They advised me to take medicine regularly. They also said to eat properly. Additionally, I was informed that the child must be kept clean, and I must also be clean.

**Q: What documentation and paperwork did they give you when you were discharged from the MLC?**

**Answer-15**

They gave me the prescription when I left the hospital. When the medication should be taken was specified in the prescription. They told me to come further for a checkup after seven days. And then she told me that she would give me medicine again.

**Q: Before you gave birth, what information did the MLC give you about what would happen if there was a complication or emergency that meant you needed to transfer to a hospital?**

**Answer-16**

They told me why I should come to the hospital. They also told me to come if there was any problem.

**Q: Did you or your baby need to be transferred to another facility either during labour or shortly after the birth? Why? Tell me about that experience. How did you feel?**

**Answer-17**

I did not go to any other hospital after having my baby. I just came to this hospital. My baby is fine, and so am I.

**Q: How did you make the journey from your home to the MLC? What would have made their journey easier for you?**

**Answer-18**

After the labor pain started, Amina Khala went to fetch me. I came on foot with them to the hospital. It would have been better to have a transport system. There was no transport facility, so I walked with difficulty.

**Q: Would you give birth at MLC again in future, or recommend the MLC to a friend or relative? Why?**

**Answer-19**

I will tell people around me that I came to this hospital. As I like it, they can go. They will be fine too.

**Q: What are the things that could have been improved further? Please describe three main things you would suggest for improvement.**

**Answer-20**

When people have labor pains, they have to walk to this hospital. It is very difficult. If there was a transport system, it would be convenient for people to come here. Due to the hilly area, vehicles cannot move. Therefore, the road has to be repaired.

**Q: What is it about the MLC that makes it different from other health facilities where women can give birth?**

**Answer-21**

I like the service at this hospital, so I came here instead of going to other hospitals.

**Q: How did the midwives make you feel respected?**

**Answer-22**

The midwives treated me well. They treated me the way I liked. That is why I like this hospital.

**Q: How did the midwives encourage you to ask questions and ask for what you needed?**

**Answer-23**

The midwives took good care of me.

**Q: How did the midwives encourage you to make your own decisions about your care?**

**Answer-24**

The midwives told me to breastfeed the baby for six months. They forbade me from eating other foods before the age of six months.
