# Supplementary material for: Midwife-led birthing centre in the humanitarian setup: An experience from the Rohingya camp, Bangladesh
Source: PLOS Glob Public Health. 2024 Dec 10;4(12):e0004033. doi: 10.1371/journal.pgph.0004033 (PMC11630605; doi:10.1371/journal.pgph.0004033)
Supplement: S6 Data — (DOCX) [file pgph.0004033.s011.docx]

**IDI-04: Mrs. Samira, Cox’sbazar**

**Q: Tell me about your most recent birth at (name of MLC).**

**Answer-1**

The name of this hospital is RTMI UNFPA.

**Q: When was it? Did you have a son or a daughter?**

**Answer-2**

My baby was delivered yesterday. I gave birth to a baby boy.

**Q: Was it your first birth? If not, where did you give birth before?**

**Answer-3**

This is my fourth child. Of the previous three babies, one was born in Myanmar, one at Camp-3, and another at home.

**Q: How did you hear about the MLC and why did you choose it?**

**Answer-4**

It is good to hear about this hospital from a volunteer khala. So I came to this hospital to give birth a baby.

**Q: What did you like about the MLC?**

**Answer-5**

 There are no men in the hospital. All the service providers are women here. I like all of them. This is why I like it here.

**Q: What did you like about the staff of the MLCs? ( feel comfortable to share things or ask questions)**

**Answer-6**

They tested me and checked me. After the test, they told me about the report. They spoke about whether I was feeling good or not. I liked these.

**Q: How did they involve you and your family in decisions about your care?**

**Answer-7**

I asked the midwives to put me on the bed during delivery. They put me in the middle of the bed and delivered me. I liked it very much.

**Q: In what ways did the MLC respect your needs? (probe for things like: birth partners, language, respect for cultural traditions that are important to the woman)**

**Answer-8**

. The midwives understood everything I said. I like it very much. They are all good.

**Q: What or who helped you to pay the costs of accessing care? (probe as appropriate for: user fees, transport costs, food and accommodation for self and family members, medicine costs, equipment costs (e.g. sanitary pads)**

**Answer-9**

I didn't have to spend any money here. They gave me food, sanitary pads, and everything. They gave me soap, a bucket, everything.

**Q: Would you recommend the MLC services to other women? If yes or no why?**

**Answer-10**

I will go home and say to others, "Those who are pregnant should go to the hospital and deliver. You don't need any money. They work very well in the hospital."

**Q: What are three main things to be changed for better services in future?**

**Answer-11**

Everything is good in this hospital. But the bathroom is far away. It was very difficult to go to the bathroom.

**Q: Do you think the MLC has all the health workers, materials and equipment it needs to provide high quality childbirth services? What should be done to make it better in future?**

**Answer-12**

I like everything. Midwives are all good. There is no need for changes.

**Q: What did the midwives do to make you feel confident that they knew how to do their job well?**

**Answer-13**

The midwives are all good here. They are just like my family. I can tell them everything on my mind. When they check me up, I feel like I'm at home rather than in the hospital.

**Q: What did the midwives do to make you feel confident in your own ability to give birth safely and care for your baby?**

**Answer-14**

They cleaned my baby nicely, cut the umbilical cord, and told me to breastfeed.

**Q: What documentation and paperwork did they give you when you were discharged from the MLC?**

**Answer-15**

They explained all the documents to me. They explained to me what medicines to take. They will pick me up in the car after explaining everything while leaving here.

**Q: Before you gave birth, what information did the MLC give you about what would happen if there was a complication or emergency that meant you needed to transfer to a hospital?**

**Answer-16**

I came here after having labor pains. My baby was born within 30 minutes of my arrival at the hospital. So they didn't tell me anything like that.

**Q: Did you or your baby need to be transferred to another facility either during labour or shortly after the birth? Why? Tell me about that experience. How did you feel?**

**Answer-17**

I did not need anywhere to go after having children.

**Q: How did you make the journey from your home to the MLC? What would have made their journey easier for you?**

**Answer-18**

A volunteer khala brought me here in an ambulance. I didn't have to pay anything.

**Q: Would you give birth at MLC again in future, or recommend the MLC to a friend or relative? Why?**

**Answer-19**

I will come again to this hospital for services. I will tell all my relatives that this hospital is good.

**Q: What are the things that could have been improved further? Please describe three main things you would suggest for improvement.**

**Answer-20**

I like everything here. But a change is needed. If anyone comes here for service, I request that you try to provide service as soon as possible.

**Q: What is it about the MLC that makes it different from other health facilities where women can give birth?**

**Answer-21**

I like that every single person here is a woman.

**Q: How did the midwives make you feel respected?**

**Answer-22**

The midwives valued my opinion. I was able to tell them my thoughts.

**Q: How did the midwives encourage you to ask questions and ask for what you needed?**

**Answer-23**

I could easily question them. I asked them in my own language.

**Q: How did the midwives encourage you to make your own decisions about your care?**

**Answer-24**

They told me how to take care of the baby and myself. They asked me to vaccinate the baby. They told me to feed the baby well. They said everything.
