# Supplementary material for: Midwife-led birthing centre in the humanitarian setup: An experience from the Rohingya camp, Bangladesh
Source: PLOS Glob Public Health. 2024 Dec 10;4(12):e0004033. doi: 10.1371/journal.pgph.0004033 (PMC11630605; doi:10.1371/journal.pgph.0004033)
Supplement: S7 Data — (DOCX) [file pgph.0004033.s012.docx]

**IDI-5: Halima Khatun, AUD-20221226-( WA0002+WA0001), Cox’sbazar**

**Q: Tell me about your most recent birth at (name of MLC).**

**Answer-1**

The name of this hospital is RTMI Hospital.

**Q: When was it? Did you have a son or a daughter?**

**Answer-2**

I delivered a baby two days ago. It's a baby girl.

**Q: Was it your first birth? If not, where did you give birth before?**

**Answer-3**

This child is my second child. The previous baby was also born in the hospital.

**Q: How did you hear about the MLC and why did you choose it?**

**Answer-4**

I heard about this service center from Samuda khala. I also gave birth to my previous child here. I like it here, so I will come again.

**Q: What did you like about the MLC?**

**Answer-5**

 I like everyone here. I like all the doctors and midwives here.

**Q: What did you like about the staff of the MLCs? ( feel comfortable to share things or ask questions)**

**Answer-6**

After the delivery, the midwives stitched me up. After that, I had no more pain. I like it very much.

**Q: How did they involve you and your family in decisions about your care?**

**Answer-7**

Before I had the baby, the doctor asked me what method of delivery I wanted. After I told them, they also asked my husband what procedure he wanted. Then they took me to the delivery room and gave me iodine in the birth canal.

**Q: In what ways did the MLC respect your needs? (probe for things like: birth partners, language, respect for cultural traditions that are important to the woman)**

**Answer-8**

Those who are midwives here understand my language.

**Q: What or who helped you to pay the costs of accessing care? (probe as appropriate for: user fees, transport costs, food and accommodation for self and family members, medicine costs, equipment costs (e.g. sanitary pads)**

**Answer-9**

It costs me nothing to give birth here. They brought me here in an ambulance when my labor pains began. I got my delivery after coming here. After delivery, they gave me a bag, a blanket, soap, a bucket, and everything else. They have served me beautifully. They also fed me well.

**Q: Would you recommend the MLC services to other women? If yes or no why?**

**Answer-10**

I would encourage other women to come here for service. This RTMI hospital is very good and has served us a lot. They took great care of me during the delivery of my baby. I will tell them, "You go to this hospital to deliver the baby."

**Q: What are three main things to be changed for better services in future?**

**Answer-11**

I like it very much here. I have been well taken care of. But I didn't like one thing. There is no bathroom here. Delivery patients like us have to walk a long distance to use the bathroom. Besides, I like everything here.

**Q: Do you think the MLC has all the health workers, materials and equipment it needs to provide high quality childbirth services? What should be done to make it better in future?**

**Answer-12**

I like everything here. Everyone here has served me a lot. When my baby cried at night, they took care of her. Everything is good except for the bathroom.

**Q: What did the midwives do to make you feel confident that they knew how to do their job well?**

**Answer-13**

The midwives respected me after the baby was delivered; their manners were very nice. I like it very much.

**Q: What did the midwives do to make you feel confident in your own ability to give birth safely and care for your baby?**

**Answer-14**

When I gave birth, the midwives delivered the baby with care. After the baby was delivered, they cleaned it nicely, wrapped it in a blanket, and gave it to me to breastfeed. I love it when they take care of me like this.

**Q: What documentation and paperwork did they give you when you were discharged from the MLC?**

**Answer-15**

They gave me an ANC card after my son was born. They have taken both my husband's and my smart cards in order to issue the child's certificate. The midwives clearly explained when I should take the medication.

**Q: Before you gave birth, what information did the MLC give you about what would happen if there was a complication or emergency that meant you needed to transfer to a hospital?**

**Answer-16**

They told me that if the delivery is not good here, they will send me elsewhere. It didn't take long for me to deliver the baby. My baby was delivered within 15–20 minutes.

**Q: Did you or your baby need to be transferred to another facility either during labour or shortly after the birth? Why? Tell me about that experience. How did you feel?**

**Answer-17**

I didn't have to move anywhere. I gave birth before that.

**Q: How did you make the journey from your home to the MLC? What would have made their journey easier for you?**

**Answer-18**

I came to this service center by hospital ambulance from home. They will drop me back home in the ambulance.

**Q: Would you give birth at MLC again in future, or recommend the MLC to a friend or relative? Why?**

**Answer-19**

I will come again to this service center for service. I will tell my family and those around me, "You go to RTMI Hospital; you will get good service there. I also delivered two babies there."

**Q: What are the things that could have been improved further? Please describe three main things you would suggest for improvement.**

**Answer-20**

There is no need to change. I did not like the washroom arrangement here. The washroom is too far away for the delivery patient. It would be better for the delivery patient if the washroom was nearby. Otherwise, we have to go far, for which we suffer.

**Q: What is it about the MLC that makes it different from other health facilities where women can give birth?**

**Answer-21**

I sometimes forget about the day of the checkup; I don't remember. Samuda Khala, the volunteer here, brought me. After I came here, the midwives checked me up and gave me a piece of paper so that when I see that piece of paper, I will remember the next checkup. I like it very much.

**Q: How did the midwives make you feel respected?**

**Answer-22**

When I arrived, the doctors and midwives asked if I was okay. I loved it when a doctor asked me so nicely.

**Q: How did the midwives encourage you to ask questions and ask for what you needed?**

**Answer-23**

The midwives asked me questions in my own language. I do not understand any language except Rohingya. I loved how much they were able to speak to me in my language.

**Q: How did the midwives encourage you to make your own decisions about your care?**

**Answer-24**

Midwives advised me to keep the baby clean. They told me to eat good, nutritious food, take care of myself, and breastfeed the baby.
