# Supplementary material for: Midwife-led birthing centre in the humanitarian setup: An experience from the Rohingya camp, Bangladesh
Source: PLOS Glob Public Health. 2024 Dec 10;4(12):e0004033. doi: 10.1371/journal.pgph.0004033 (PMC11630605; doi:10.1371/journal.pgph.0004033)
Supplement: S8 Data — (DOCX) [file pgph.0004033.s013.docx]

**IDI-06: Fatema Khatun AUD-20221226-( WA0004+WA0003), Cox’sbazar**

**Q: Tell me about your most recent birth at (name of MLC).**

**Answer-1**

My baby was delivered at the RTMI hospital.

**Q: When was it? Did you have a son or a daughter?**

**Answer-2**

I gave birth to a baby boy. Five days ago, the baby was born.

**Q: Was it your first birth? If not, where did you give birth before?**

**Answer-3**

This is my second baby. The previous child was born at home.

**Q: How did you hear about the MLC and why did you choose it?**

**Answer-4**

CSW Khala said the midwives here test the patients. So I came here. After I came here, they checked my abdomen. I like their speech. So I came here a few times for checkups, then delivered the baby.

**Q: What did you like about the MLC?**

**Answer-5**

Here they look after the patients, look after the children, and comfort them beautifully. I like them.

**Q: What did you like about the staff of the MLCs? ( feel comfortable to share things or ask questions)**

**Answer-6**

They treated me well and delivered beautifully and with care. I like it very much. Patients are scolded at other hospitals.

**Q: How did they involve you and your family in decisions about your care?**

**Answer-7**

When I came here for delivery, they made me sit if I wanted to sit, made me walk, and told my husband about different methods and medicines to not have the baby too early. I don't know what to do if the baby has a problem. I learned numerous guidelines from them, in addition to how to care for the child. I can learn a lot from them.

**Q: In what ways did the MLC respect your needs? (probe for things like: birth partners, language, respect for cultural traditions that are important to the woman)**

**Answer-8**

Some of the Apas here understand what I'm saying. And the CSW aunts have explained to the midwives who are present if they don't understand what I'm saying. Then they understood.

**Q: What or who helped you to pay the costs of accessing care? (probe as appropriate for: user fees, transport costs, food and accommodation for self and family members, medicine costs, equipment costs (e.g. sanitary pads)**

**Answer-9**

It didn't cost me any money to deliver the baby here. Here, they deliver for free and also give us medicine and food. We do not have to pay anything for delivery here. I didn't even have to buy sanitary pads; they provided them. They also gave me a bucket and soap. I didn't even need to hire a vehicle to come here. They brought me here in an ambulance, and when I left, they also transported me by ambulance.

**Q: Would you recommend the MLC services to other women? If yes or no why?**

**Answer-10**

The facilities that I got from this hospital are so good that if more people come and their deliveries go well and they also get these facilities, I will tell my neighbors to come here.

**Q: What are three main things to be changed for better services in future?**

**Answer-11**

I like everything here. But there is no bathroom for urinating, toileting, or showering. We have to go outside to go to the bathroom. This makes me feel worse. Another thing I feel bad about. It is difficult to call someone in an emergency due to a bad network. As a result, I frequently have to attempt to contact someone.

**Q: Do you think the MLC has all the health workers, materials and equipment it needs to provide high quality childbirth services? What should be done to make it better in future?**

**Answer-12**

Everything is here. Nothing else is needed. The service I have received here is great. I would not have had such an advantage in Burma.

**Q: What did the midwives do to make you feel confident that they knew how to do their job well?**

**Answer-13**

After I came here, the midwives did my checkup. If I told them about any stomach problems, they helped me. My pain is less. They asked me how the pain was. They cheerfully ask me everything; I like it. They respectfully ask me what's wrong with me and whether I'm in pain. They comforted me when I was in great pain. They took good care of me, served me. That's why I came here for a checkup. I come here because I like the services; otherwise, I wouldn't have come.

**Q: What did the midwives do to make you feel confident in your own ability to give birth safely and care for your baby?**

**Answer-14**

My baby didn't cry after birth. The midwives carefully examined my baby, put their hands on the baby's chest, and then my baby cried. Then put the baby in my arms.

**Q: What documentation and paperwork did they give you when you were discharged from the MLC?**

**Answer-15**

They gave me medicine while leaving here. They gave me medicine for pain and energy. The documents required for the child have been provided. Then they took me home by vehicle.

**Q: Before you gave birth, what information did the MLC give you about what would happen if there was a complication or emergency that meant you needed to transfer to a hospital?**

**Answer-16**

I was told by the midwives that they would refer me if there were any problems with the delivery, or no delivery, or if there were any problems with the baby.

**Q: Did you or your baby need to be transferred to another facility either during labour or shortly after the birth? Why? Tell me about that experience. How did you feel?**

**Answer-17**

I didn't have to go anywhere else.

**Q: How did you make the journey from your home to the MLC? What would have made their journey easier for you?**

**Answer-18**

I did not arrive here by renting any transportation. Here I am in the hospital ambulance. I used a vehicle to get to the checkup and to return home.

**Q: Would you give birth at MLC again in future, or recommend the MLC to a friend or relative? Why?**

**Answer-19**

I will come here again. I like it here; their way of communicating is very good. I didn't have to pay anything here; everything is done here for free; in Burma, I had to pay expenses there. Since it is free and the government pays the expenses here, my relatives will come here happily. The midwives here speak very nicely; there is really no problem here. They can come.

**Q: What are the things that could have been improved further? Please describe three main things you would suggest for improvement.**

**Answer-20**

There is nothing to do. Everything is good here.

**Q: What is it about the MLC that makes it different from other health facilities where women can give birth?**

**Answer-21**

Other hospitals have male doctors, but here all the doctors and midwives are female; there are no boys. I like it very much. If there are men, I feel shy. This is why I like it here. So I delivered my baby here.

**Q: How did the midwives make you feel respected?**

**Answer-22**

The midwives spoke to me nicely after coming here. They asked me where I felt bad or what was wrong with me. The midwives here understand our joys and sorrows well. Talking to them gives me peace; that's why I like it here.

**Q: How did the midwives encourage you to ask questions and ask for what you needed?**

**Answer-23**

No one asked me a question in a bad way. I wouldn't have delivered here if I did. They actually talk to me very nicely during the checkup. They delivered my baby beautifully, just the way I liked it.

**Q: How did the midwives encourage you to make your own decisions about your care?**

**Answer-24**

After having a baby, they told me not to bathe the baby for three days. Besides, they told me to keep the baby neat and clean. She also asked me to vaccinate the baby, breastfeed properly until the baby was six months old, and keep the baby clean. This is what they explained to me.
