# Supplementary material for: Midwife-led birthing centre in the humanitarian setup: An experience from the Rohingya camp, Bangladesh
Source: PLOS Glob Public Health. 2024 Dec 10;4(12):e0004033. doi: 10.1371/journal.pgph.0004033 (PMC11630605; doi:10.1371/journal.pgph.0004033)
Supplement: S9 Data — (DOCX) [file pgph.0004033.s014.docx]

**IDI-07: Mrs. Mahmuda, Cox’sbazar**

**Q: Tell me about your most recent birth at (name of MLC).**

**Answer-1**

The name of this hospital is RTMI Hospital.

**Q: When was it? Did you have a son or a daughter?**

**Answer-2**

I had a baby on November 24, 2022, at 3:05 p.m. I gave birth to a baby boy.

**Q: Was it your first birth? If not, where did you give birth before?**

**Answer-3**

This is my third child. The previous two children were born in Burma.

**Q: How did you hear about the MLC and why did you choose it?**

**Answer-4**

I heard about this hospital through my younger sister-in-law. Also, everyone agreed that the midwives at the RTMI UNFPA hospital are much better than those at other hospitals. They test the patients very well. That's why I came here.

**Q: What did you like about the MLC?**

**Answer-5**

I like everything about this hospital. Because the midwives allowed my sister to accompany me as an attendant and checked me on a regular basis, they massaged my back. They explained everything to me, including my family. After my baby was born, the baby was put to my chest, the baby was cleaned, the umbilical cord was cut, and medicine was given to me. Also, they took permission from me before doing anything. I like everything about them, and the hospital rooms are wide.

**Q: What did you like about the staff of the MLCs? ( feel comfortable to share things or ask questions)**

**Answer-6**

The midwives explained to me and my family very well that my baby and I are fine, and delivery here does not cost anything. They advised me to tell them if there were any other problems. And every time the midwives checked me up, they told me my baby was fine, and so was I. Those who are cleaners are also very good here. The doctor came here during the day and checked me up, as well as my baby. If I wanted to bring something from outside, they would bring it if I asked the brothers here. I like everything about the staff here.

**Q: How did they involve you and your family in decisions about your care?**

**Answer-7**

They talked to me nicely and tested me right after I came here. They tested me, and everything was fine. They were asking me and my family to pray and listen to what they said. I was told about family planning and whether I should take the pill. They can also refer me if there is a problem with me or my baby, in which case they will provide me transportation and a midwife will accompany me.

**Q: In what ways did the MLC respect your needs? (probe for things like: birth partners, language, respect for cultural traditions that are important to the woman)**

**Answer-8**

They listened to what I said and asked about my pros and cons. I didn't want to exercise. They said that if I exercised, the delivery would be quicker. Before testing me, they pulled back the curtain. They could not understand some of my words, but they understood through Khala. They didn't abuse me; they didn't even get angry with me.

**Q: What or who helped you to pay the costs of accessing care? (probe as appropriate for: user fees, transport costs, food and accommodation for self and family members, medicine costs, equipment costs (e.g. sanitary pads)**

**Answer-9**

Delivery or ANC—any service at the RTMI hospital is free of charge. The midwives gave me medicine, pads, the mama kit, and kit 2A. I didn't have to pay any money for those. They did not take any money from my inventory.

**Q: Would you recommend the MLC services to other women? If yes or no why?**

**Answer-10**

I will definitely tell others to visit this hospital. Because there are many facilities available here. They bring us from home by ambulance, so we don't have to walk; again, they deliver us home. The midwives did a thorough checkup on us and pulled the curtain before the checkup.

**Q: What are three main things to be changed for better services in future?**

**Answer-11**

I like everything about them. But it would have been better if the washroom was closer. One advantage of being away is that it doesn't smell.

**Q: Do you think the MLC has all the health workers, materials and equipment it needs to provide high quality childbirth services? What should be done to make it better in future?**

**Answer-12**

I think all the ingredients are there to deliver. I have heard that children should be referred to Hope or another hospital if they have any problems. It would be great if this hospital had medicine for children. If there is a facility to do ultrasonography here, it would have a lot of benefits.

**Q: What did the midwives do to make you feel confident that they knew how to do their job well?**

**Answer-13**

Midwives talk to us very well. With them, we can say everything; midwives don't get angry. If I don't understand something, they explain it to me and do a good checkup. I heard from my sister-in-law and other people that they are very good and provide good service.

**Q: What did the midwives do to make you feel confident in your own ability to give birth safely and care for your baby?**

**Answer-14**

They delivered me while wearing gloves to prevent infection. They drew the curtains, provided a cloth to wipe the baby. After I gave birth, they put the baby on my chest, cut the baby's umbilical cord, gave me medicine, and measured the baby's weight.

**Q: What documentation and paperwork did they give you when you were discharged from the MLC?**

**Answer-15**

While I was leaving here, they gave me my clearance and the birth certificate for the baby. They gave me an ANC card and a Data card. They also explained to me about the medicine.

**Q: Before you gave birth, what information did the MLC give you about what would happen if there was a complication or emergency that meant you needed to transfer to a hospital?**

**Answer-16**

Before I gave birth in this hospital, the midwives told me that if I or my baby had any problems, they would refer us, and they would provide the ambulance service as well. In that case, a midwife will also go with me. CHW Khala will also go with me and stay.

**Q: Did you or your baby need to be transferred to another facility either during labour or shortly after the birth? Why? Tell me about that experience. How did you feel?**

**Answer-17**

I didn't have to transfer anywhere else. I was in this hospital.

**Q: How did you make the journey from your home to the MLC? What would have made their journey easier for you?**

**Answer-18**

I came from my house to this hospital by ambulance. CHW Khala also came with me. If there had not been mobile network problem, I could have come earlier.

**Q: Would you give birth at MLC again in future, or recommend the MLC to a friend or relative? Why?**

**Answer-19**

I will not have any more children. If so, then I will come to the Camp U Ext hospital. I will advise my relatives and friends to come here too. Because very good service is provided here. There are no men in this hospital.

**Q: What are the things that could have been improved further? Please describe three main things you would suggest for improvement.**

**Answer-20**

Everything is good in this hospital.

**Q: What is it about the MLC that makes it different from other health facilities where women can give birth?**

**Answer-21**

This hospital has a transportation service that can bring us here for check-ups and also deliver us home. Midwives do checkups very well, pulling back the curtain before doing the checkup. No one lets men in without permission. And CHW Khalas are always here.

**Q: How did the midwives make you feel respected?**

**Answer-22**

Missing

**Q: How did the midwives encourage you to ask questions and ask for what you needed?**

**Answer-23**

Missing

**Q: How did the midwives encourage you to make your own decisions about your care?**

**Answer-24**

Missing
