# Supplementary material for: Midwife-led birthing centre in the humanitarian setup: An experience from the Rohingya camp, Bangladesh
Source: PLOS Glob Public Health. 2024 Dec 10;4(12):e0004033. doi: 10.1371/journal.pgph.0004033 (PMC11630605; doi:10.1371/journal.pgph.0004033)
Supplement: S10 Data — (DOCX) [file pgph.0004033.s015.docx]

**IDI-8: Aneta Begum, Cox’sbazar**

**Q: Tell me about your most recent birth at (name of MLC).**

**Answer-1**

The name of this hospital is RTMI Hospital.

**Q: When was it? Did you have a son or a daughter?**

**Answer-2**

This baby was born at the RTMI Camp 4 Ext Hospital. I delivered my baby on November 13, 2022, at 1:47 PM. This is a baby boy.

**Q: Was it your first birth? If not, where did you give birth before?**

**Answer-3**

This is my fifth child. This child was born at the RTMI Camp 4 Ext hospital.

**Q: How did you hear about the MLC and why did you choose it?**

**Answer-4**

I heard about this hospital through CHW Khala. I like this hospital better than other hospitals. When I used to come here for an ANC checkup, the midwives used to talk to me very well and check me very well.

**Q: What did you like about the MLC?**

**Answer-5**

The ANC checkup in this hospital is very good, and children are vaccinated here. The rooms in this hospital are very big. If we are admitted to this hospital, they give us food on time.

**Q: What did you like about the staff of the MLCs? ( feel comfortable to share things or ask questions)**

**Answer-6**

The midwives at RTMI Camp:4 Ext Hospital are fantastic; they thoroughly test us and converse with us. They pulled the curtain while we were testing. Doctor Ma'am is also very good. She went over the advantages of a hospital delivery versus a home delivery with me. The cleaner here is also very good. They told me where to throw the blood and stool that came out during my delivery. And everyone in this hospital treated me and my family very well.

**Q: How did they involve you and your family in decisions about your care?**

**Answer-7**

The midwives asked for my permission before testing me, and during the labor pains, they told me to exercise as much as I wanted. They explained the advantages of giving birth in this hospital. Along with this, they also informed me about the equipment available in the hospital for a normal delivery. They also told me that if there is a problem with me or my baby, there is a referral facility; there is an ambulance facility if there is a referral, and in that case a midwife will also accompany me. Furthermore, they informed me and all my family about family planning.

**Q: In what ways did the MLC respect your needs? (probe for things like: birth partners, language, respect for cultural traditions that are important to the woman)**

**Answer-8**

The midwives were very respectful of my needs. I did not like lying on the labor table. Then they told me to sit on the chair. But when I wanted to sleep downstairs, they arranged for me to sleep downstairs. I had a lot of pain in my back, so they gave me a massage. When I told them to take Depo, they arranged it. They understood everything I said.

**Q: What or who helped you to pay the costs of accessing care? (probe as appropriate for: user fees, transport costs, food and accommodation for self and family members, medicine costs, equipment costs (e.g. sanitary pads)**

**Answer-9**

The delivery to RTMI Camp:4 Ext Hospital is free. Neither the midwives nor any of the staff took any money from us. They gave us the Mama Kit, the Hygiene Kit, and Kit 2A, but they didn't take any money.

**Q: Would you recommend the MLC services to other women? If yes or no why?**

**Answer-10**

I will encourage other women to seek services here. Because the midwives and CHW aunties here are very good. Other hospitals do not allow anyone to stay with the patient. But here, my mother was allowed to stay with me and gave me my baby on my chest after delivery. They cleaned my baby's nose and mouth thoroughly and cut the umbilical cord, but they took my permission before doing anything. They gave me food on time. The best part is that they pull the curtain before the checkup. The midwives were right by our sides, helping us with the exercises and explaining everything to me. Also, they give us a lot.

**Q: What are three main things to be changed for better services in future?**

**Answer-11**

To do better in the future, it would be better if the washroom here was near the delivery room.

**Q: Do you think the MLC has all the health workers, materials and equipment it needs to provide high quality childbirth services? What should be done to make it better in future?**

**Answer-12**

I think this hospital has everything required because the midwives, doctors, and CHWs here do the tests very well. And when I came for the ANC checkup, I had a little problem; I used to sit for a long time to get treatment. As there were many people, it took them a long time to see the patients. It would be better if they saw patient sooner. And it would have been better if computerized services had been arranged here.

**Q: What did the midwives do to make you feel confident that they knew how to do their job well?**

**Answer-13**

The midwife talked to me for a long time. They explained everything about the hospital and the services available to me in great detail. When I came for my ANC checkup, they did my checkup very well and explained everything to me. They did each job very well.

**Q: What did the midwives do to make you feel confident in your own ability to give birth safely and care for your baby?**

**Answer-14**

The midwives said my baby was fine, and so was I. They let me hear the breathing sound of my baby. During the delivery, I peed on the delivery bed, but they took it very easy. The midwives cleaned my baby's nose and throat after delivery, then cut the umbilical cord, applied medicine to the umbilical cord, and injected the baby. In addition, the child was given clothes to wipe.

**Q: What documentation and paperwork did they give you when you were discharged from the MLC?**

**Answer-15**

They gave me a clearance paper when I was discharged from this service center. My child's birth certificate, ANC card, and blood test results were also given to me. B/C wrote about my data card; they gave me that too.

**Q: Before you gave birth, what information did the MLC give you about what would happen if there was a complication or emergency that meant you needed to transfer to a hospital?**

**Answer-16**

The midwives told me my baby and I were fine. If there is a problem, they will be taken to the hospital by ambulance, accompanied by a midwife. Another CHW will accompany me.

**Q: Did you or your baby need to be transferred to another facility either during labour or shortly after the birth? Why? Tell me about that experience. How did you feel?**

**Answer-17**

I didn't have to go anywhere. I was here.

**Q: How did you make the journey from your home to the MLC? What would have made their journey easier for you?**

**Answer-18**

I called CHW Fatima Khala and after she came, she called an ambulance. Then we came to the hospital. When I called the ambulance, the phone was not working; the network problem was too much. It would be better if the phone call could be answered immediately.

**Q: Would you give birth at MLC again in future, or recommend the MLC to a friend or relative? Why?**

**Answer-19**

I will deliver at this hospital again and tell everyone around me to come here. Because there are many good services available here. They don't have to walk to the hospital. Everyone at the hospital is very good. After delivery, the baby can be vaccinated here; there is no need to go to another place.

**Q: What are the things that could have been improved further? Please describe three main things you would suggest for improvement.**

**Answer-20**

I don't know. I like everything here.

**Q: What is it about the MLC that makes it different from other health facilities where women can give birth?**

**Answer-21**

Other hospitals don't allow anyone to stay with patients, but here they do. Here, we are transported by the CHW aunts to the hospital and then transported by them to our house. Food is served here on time. Moreover, they pull the curtain during the checkup.

**Q: How did the midwives make you feel respected?**

**Answer-22:** Missing

**Q: How did the midwives encourage you to ask questions and ask for what you needed?**

**Answer-23:** Missing

**Q: How did the midwives encourage you to make your own decisions about your care?**

**Answer-24:** Missing
