# Supplementary material for: Midwife-led birthing centre in the humanitarian setup: An experience from the Rohingya camp, Bangladesh
Source: PLOS Glob Public Health. 2024 Dec 10;4(12):e0004033. doi: 10.1371/journal.pgph.0004033 (PMC11630605; doi:10.1371/journal.pgph.0004033)
Supplement: S11 Data — (DOCX) [file pgph.0004033.s016.docx]

**IDI- 9_Cox’sBazar_Rokhsana**

**Q: Tell me about your most recent birth at (name of MLC).**

**Answer-1**

My baby was born in the Rohingya Camp Hospital.

**Q: When was it? Did you have a son or a daughter?**

**Answer-2**

My baby was born four months ago. I gave birth to a baby boy.

**Q: Was it your first birth? If not, where did you give birth before?**

**Answer-3**

This is my fifth child. Previously my other childs were born at home.

**Q: How did you hear about the MLC and why did you choose it?**

**Answer-4** **:** I heard about it from neighbor.

**Q: What did you like about the MLC?**

**Answer-5** **:** They took care of me a lot. They still take update about me and my baby time to time.

**Q: What did you like about the staff of the MLCs? ( feel comfortable to share things or ask questions)**

**Answer-6** **:** They instructed me well throughout the process. They taught me some exercises which helped me to deliver the baby.

**Q: How did they involve you and your family in decisions about your care?**

**Answer-7:** They talked very respectfully to my family. Informed about my health condition to my family.

**Q: In what ways did the MLC respect your needs? (probe for things like: birth partners, language, respect for cultural traditions that are important to the woman)**

**Answer-8:** Missing

**Q: What or who helped you to pay the costs of accessing care? (probe as appropriate for: user fees, transport costs, food and accommodation for self and family members, medicine costs, equipment costs (e.g. sanitary pads)**

**Answer-9**

My baby was born four months ago. Until then, I had gone to the clinic for a checkup. At the clinic, they asked me to do a blood test, gave me iron and calcium tablets. The clinic gave me iron tablets, and I bought calcium tablets myself.

**Q: Would you recommend the MLC services to other women? If yes or no why?**

**Answer-10:** Yes, I already told to my neighbors and family members to visit for any need.

**Q: What are three main things to be changed for better services in future?**

**Answer-11:** Missing

**Q: Do you think the MLC has all the health workers, materials and equipment it needs to provide high quality childbirth services? What should be done to make it better in future?**

**Answer-12:** Missing

**Q: What did the midwives do to make you feel confident that they knew how to do their job well?**

**Answer-13**

They have served me very well. Even now, midwives still call to see how I'm doing. They asked me to contact them if there was any problem.

**Q: What did the midwives do to make you feel confident in your own ability to give birth safely and care for your baby?**

**Answer-14**

They made me exercise to ease my pain during delivery. They gave me courage that me & my baby will be healthy.

**Q: What documentation and paperwork did they give you when you were discharged from the MLC?**

**Answer-15:** I don’t know much about this. May be they provided papers to my family. They gave me vaccination card for my baby.

**Q: Before you gave birth, what information did the MLC give you about what would happen if there was a complication or emergency that meant you needed to transfer to a hospital?**

**Answer-16**

In case I need blood while having a baby, I was asked to manage a blood donor in advance, and they also wrote it on the paper. They have served me a lot.

**Q: Did you or your baby need to be transferred to another facility either during labour or shortly after the birth? Why? Tell me about that experience. How did you feel?**

**Answer-17**

There were no problems during childbirth. The baby coughed only after giving birth. That's why I took the baby to the doctor at Cox's Bazar Hospital. Now the child has recovered.

**Q: How did you make the journey from your home to the MLC? What would have made their journey easier for you?**

**Answer-18**

I took a vehicle to the hospital to give birth as it is very near to my house.

**Q: Would you give birth at MLC again in future, or recommend the MLC to a friend or relative? Why?**

**Answer-19:**

I like their service. I will send everyone I know to that hospital to get services.

**Q: What are the things that could have been improved further? Please describe three main things you would suggest for improvement.**

**Answer-20:**

This hospital does not charge any delivery charges. There are many facilities in this hospital, they provide us with more services, and they deliver babies very nicely.

**Q: What is it about the MLC that makes it different from other health facilities where women can give birth?**

**Answer-21:** They still contact with me, I can call them whenever I need help about my health or my baby’s health.

**Q: How did the midwives make you feel respected?**

**Answer-22:**

They showed respect to me and my family members. They spoke to us with respect.

**Q: How did the midwives encourage you to ask questions and ask for what you needed?**

**Answer-23:** No response

**Q: How did the midwives encourage you to make your own decisions about your care?**

**Answer-24:**

They told us to vaccinate the child regularly.
