# Supplementary material for: Midwife-led birthing centre in the humanitarian setup: An experience from the Rohingya camp, Bangladesh
Source: PLOS Glob Public Health. 2024 Dec 10;4(12):e0004033. doi: 10.1371/journal.pgph.0004033 (PMC11630605; doi:10.1371/journal.pgph.0004033)
Supplement: S12 Data — (DOCX) [file pgph.0004033.s017.docx]

**IDI- 10: Rohingya Capm-4, Cox’sbazar**

**Q: Tell me about your most recent birth at (name of MLC).**

**Answer-1**

The name of this hospital is Rohinga Camp.

**Q: When was it? Did you have a son or a daughter?**

**Answer-2**

I gave birth 7 days ago. I gave birth to a baby boy.

**Q: Was it your first birth? If not, where did you give birth before?**

**Answer-3**

This is our fifth child. Of the previous four babies, two were born in hospitals and the other two at home.

**Q: How did you hear about the MLC and why did you choose it?**

**Answer-4** **:**

From my sister. I came here as my sister said their services are good & give mental support also give counseling.

**Q: What did you like about the MLC?**

**Answer-5** **:** They gave good mental support.

**Q: What did you like about the staff of the MLCs? ( feel comfortable to share things or ask questions)**

**Answer-6** **:**

From the hospital, they call us every day to know the physical condition of the baby and the mother.

**Q: How did they involve you and your family in decisions about your care?**

**Answer-7:** They informed my condition, my baby’s condition to my family. They told my family about financial preparation.

**Q: In what ways did the MLC respect your needs? (probe for things like: birth partners, language, respect for cultural traditions that are important to the woman)**

**Answer-8:** They gave me respect, talked in friendly way as I am a part of them.

**Q: What or who helped you to pay the costs of accessing care? (probe as appropriate for: user fees, transport costs, food and accommodation for self and family members, medicine costs, equipment costs (e.g. sanitary pads)**

**Answer-9**

We have spent about BDT 500. No money was required for the blood. I only gave the transportation fare to the blood donors who came.

**Q: Would you recommend the MLC services to other women? If yes or no why?**

**Answer-10:** Yes

**Q: What are three main things to be changed for better services in future?**

**Answer-11:** I don’t think any other changes are needed.

**Q: Do you think the MLC has all the health workers, materials and equipment it needs to provide high quality childbirth services? What should be done to make it better in future?**

**Answer-12:** They are already giving good services.

**Q: What did the midwives do to make you feel confident that they knew how to do their job well?**

**Answer-13**

My wife received good care from the hospital when she had the baby, which gave her courage. They have cooperated with us as far as possible.

**Q: What did the midwives do to make you feel confident in your own ability to give birth safely and care for your baby?**

**Answer-14**

Mother and baby were safe at the hospital. They treated us well.

**Q: What documentation and paperwork did they give you when you were discharged from the MLC?**

**Answer-15:**

We were not given any documents when we left the hospital. When the blood was donated to us, there was no documentation provided.

**Q: Before you gave birth, what information did the MLC give you about what would happen if there was a complication or emergency that meant you needed to transfer to a hospital?**

**Answer-16**

We were never told that there would be blood during delivery.

**Q: Did you or your baby need to be transferred to another facility either during labour or shortly after the birth? Why? Tell me about that experience. How did you feel?**

**Answer-17**

After the baby was delivered at the hospital, the body of the mother became weak due to bleeding. Then we were taken to Sadar Hospital. We were told about blood there. Three bags of blood were given.

The previous hospital did not tell us that blood would be needed. When the body becomes weak, they said that the patient can be admitted to the Sadar Hospital, and if the patient is not admitted to the hospital, the patient will become serious. Then we went there and admitted the patient and arranged for blood. We have arranged for two bags of blood, and one bag of blood has been arranged from the hospital.

**Q: How did you make the journey from your home to the MLC? What would have made their journey easier for you?**

**Answer-18**

The hospital is far from our house. They transport us from home to the hospital but do not provide transportation when we return. We have to walk from the hospital. It will be good for us if the transportation system is improved.

**Q: Would you give birth at MLC again in future, or recommend the MLC to a friend or relative? Why?**

**Answer-19:** Missing

**Q: What are the things that could have been improved further? Please describe three main things you would suggest for improvement.**

**Answer-20:** Missing

**Q: What is it about the MLC that makes it different from other health facilities where women can give birth?**

**Answer-21:** Missing

**Q: How did the midwives make you feel respected?**

**Answer-22:**

They spoke to us with respect and treated us well.

**Q: How did the midwives encourage you to ask questions and ask for what you needed?**

**Answer-23:** Missing

**Q: How did the midwives encourage you to make your own decisions about your care?**

**Answer-24:**

They told us to vaccinate the child regularly.
