# Supplementary material for: Midwife-led birthing centre in the humanitarian setup: An experience from the Rohingya camp, Bangladesh
Source: PLOS Glob Public Health. 2024 Dec 10;4(12):e0004033. doi: 10.1371/journal.pgph.0004033 (PMC11630605; doi:10.1371/journal.pgph.0004033)
Supplement: S13 Data — (DOCX) [file pgph.0004033.s018.docx]

**KII: Steph Marriott, International Midwifery Consultant, Cox’sbazar, Bangladesh**

***1.Question :First of all,I want to know if YOU can share us that how are you involve in MLBC and what is your role Actually?***

**Response:** So I am no longer working in Bangladesh and my contract finished at the end of .But before that for the last two years I was working with UNFPA in Cox’s Bazar as a Midwife mentor. So that involved providing mentorship and capacity building of Bangladeshi midwife Leaders and then I also did some work with programming so ensuring funds from the donors to the right places and ensuring we got the right amount of stuff at the right places and also some technical advisory work. So in regards to MLBC the most common place for this to happened to providing mentorship to midwife either pre existing mentorship. So for example family welfare. Since then UNFPA providing additional support to those facilities. The HRS services are for midwifes. So in some of those facilities there might be a medical operation.

***2.Question:Can you say something about Its background in Bangladesh?***

**Response:** My understanding of MLBC in Bangladesh is that there was an introduction of the education actually started in 2014 or 2015 of diploma midwife and these are not yet been deployed by the government in 2017 when there was large number of licensed midwives. So that’s how they’re ended up with a large number of midwives. There was also a government deployment system that runs nationally so there was gradual deployment of nurses or midwives . can’t remember the exact title. There is not much midwives. But there will be soon if they correct the education system.

***3.Question:If you can add something about the location or the Services of midwives Design, layout? How they get fund?***

**Response:** There are health Centers at different geographical level within a district in Bangladesh. The most hospitals are in the cities but there are also some district hospitals like here in Cox’s bazar and then there are Upazilla health Complexes. There are Union level support I believe it depends on the population. So this is the government structure and there are NGO’s and private sectors. There are some Licensed midwives who works only for the government and not for the private sector. So there aren’t necessary midwives in every sectors. The NGO’S deployed lots of midwives in the rohingya project but not any licensed midwives. There are many old midwives who are working for the government. So there should be an age limit. Some NGO’s take only diploma midwives or both nurses and midwives. So the structure of MLBC would look it really varies so in some of the health facilities are managed by the group of midwives .Sorry? which part of the question did I miss? So everyone can access the care. The government facilities should be free bt they’re not because of some medicine cost. The NGO’s and private sectors take charge for their services.

***4.Question :How do MLBC fit in with the wider health system?***

**Response:** The structures are different Between Division level and district level. So you would find MLBC in primary health facilities. The doctors are working in both district and upazilla hospitals. But most of the patients are in the district hospitals. The patients are different from regular patients who take services from the midwives.

***5.Question :Which organizations and NGOs support the MLBC?***

**Response:** There are many NGOs that support SRH. There are UN agencies such as UNFPA and UNHCR who provides health services in Cox’s Bazar. UNFPA build the foundation of the job.MSF provide a large number of midwives. There are lot of NGOs who worked for many problems. There were so many of them.

***6.Question :Can you say that how are communities get involve with MLBC?***

**Response:** So the Bangladeshi community in Cox’s bazar the majority of health facilities already existed before midwives or diploma midwives came. For the health facilities in the camps There is a Network of community healthcare who explained them about the healthcare facilities so they can take them. They Don’t see any difference Between midwives and normal nurses. They take these facilities as normal health care checkup. I think there are some national level advertisement about midwives. People see these advertisement and take services from midwives.

***7.Question:How the information can be shared among the communities and MLBC centers?***

**Response:** It is shared by community health workers, it shared by the midwives.

***8.Question: How the data management system works in MLBC?***

**Response:** I think there’s a lot of false information and duplication of information exist. My observation says there are six collectors who collects the data. The majority of the facilities using paper based recordings. The data collecting system Isn’t that good but if they replace it with computer data management system that will be a good decision. In the villages or far places from town there isn’t enough devices available there and the midwives out there they can’t use computer well that much.

***9. Question: How they ensure the supplies and equipments in MLBC?***

**Response:** :UNFPA supplies the equipments to the distributers and they distribute it all over the Bangladesh. The truth is they not ensure. There are huge delays of those equipments. It’s not well planned or well procrasted. There is a huge gap for medicines and equipments. If they can produce them locally then in the time of need we can use them.

***10. Question: Can you explain what other staffs do except the midwives in MLBC?***

**Response:** So there are cleaners and security guards out there. There is also a medical officer for the patients.

It all varies from facilities to facilities of its capacity. The big facilities need more workers and there are more varieties of staffs.

***11. Question: What is the role of a medical officer?***

**Response:** The medical officers provide services for the pregnant patients. Also they guide the midwives and nurses out there.

***12. Question: Who is in charge of MLBC?***

**Response:** There is a specialized midwife. She’s a diploma midwife actually. She’s the In charge of the project. She guides other midwives.

There is also a medical officer who will lead some of the projects. But mostly the midwife is the in charge.

***13.Question:How the in-charge midwife is different from the other midwives out there in Bangladesh?***

**Response:** Mostly the in charge midwife is specialized. She provides education and guidance to the other midwives. And normal midwives provides medical facilities to the patients.

***14.Question: Do you think the infrastructures need to be changed in MLBC?***

**Response:** There’s no need to change They need to ensure that there has ro be enough instruments and infrastructure out there. We need them very badly all over the country. The supply chain needs to be improve.

***15.Question:What kind of midwives MLBC should take? What are the qualifications?***

**Response:** They need to be skilled. They should have a diploma degree. They should have basic knowledge about their work. They need to have patience. They need to behave well with their patients.

16.Question:Can you say how the service provided by MLBC is evidence based?

**Response:** It is evidence based because we prove ICM standard midwives

***17.Question:Can you say some factor about how the MLBC can give High quality care?***

**Response:** Mentorship facility is high quality care. We need enough medicine and logistics. The infrastructures should be good. The staffs need to be well trained. They should not do long shifts. They should behave well with their doctors whom they’re working.

***18.Question:How the data management system care can be assured or evaluated?***

**Response:** For NGOs there are quality managers. Who ensures the data’s.

***19. Question: What issues need to be emphasized for effective coordination and partnership between midwife-run delivery centers and other types of health facilities? Give examples of how this can be improved.***

**Response:** Their work has been well explained to them. Basically, we need their cooperation most of all through the orientation of UHFPO sirs. If the manager is good, everything will work well. You say the midwife, the nurse says everyone will work properly. Orientation is very necessary to minimize. Also monitoring and mentoring is very important. We have seen that where the manager is good, everything is going well.

***20. Question:How do midwife-run birth centers engage with the community?***

**Response:** We send midwives to different workshops and seminars so that they can directly connect with people. Also we tell them what we teach them in training so that they share with people close to them. Also we advertise through mass media. Television Radio We also inform everyone about midwife services through mobile phones.

***21. Question: How does the government support midwife-run centers (what is the government's position in this regard?)***

**Response:** The government is fully supporting this. Also, the service has improved due to the government's support in late care. Orientation is being done by the UHFPO for the work of midwives. Also, we are discussing with the government so that midwives can work with full support. Honorable Prime Minister is giving a lot of importance to mother and child services. But due to the small number, we cannot ensure it everywhere.

***22. Question: How would these maternity care centers do if they were to fully meet the needs of the community?***

**Response:** Union sub-centres should have all the equipment and supporting nurses to help the midwives. Midwives cannot work alone in the sub-centers. They should have their helping nurses and darwan medicine ready. We want to be able to take care of the pregnant mothers at their doorsteps. For this, we should have improved the Union sub center first. Besides, they waste a whole day. Which is very difficult for a mother. And only if these things are improved, we will be able to provide good services to the community.

***23. Question: What factors help women trust the services of these maternity care centers?***

**Response:** Because midwives are young, many people think that they know less about childbirth. So we tell them to always be by the side of mothers. They should not be alone. They take care of their movements before delivery. As a result, mothers trust them more. by doing Midwives also provide more facilities to mothers so that they feel safe. Good service and good behavior increases the trust of mothers.

***24. Question: How Referral Management Works What if this works well?***

**Response:** Sub-centers have doctors in the morning but not in the afternoon. So midwives should be there with initial management. If they can manage the medication of PPH then there is no need for referral system. Referral can be done. But we have not yet established the referral system properly everywhere. If they are not able to handle all the medicines properly then it becomes difficult. If there is UNFPO this is not a problem. We are not yet able to provide this facility at the Union level. We hope very soon. We will take care of this In this case we need help.

***25. Question: How to integrate referral communication channels into the existing health system What an ideal referral system would look like (how to communicate with all levels of services through information sharing, how to inform service users about the referral system?)***

**Response:** We don't need referral at the initial stage. We wear the referral chain knowingly. It is step by step. First at the union level then at the district level then can refer to the medical college as well. Doctors make referrals to midwives.

***26. Question: What issues need to be emphasized for effective coordination and partnership between midwife-run delivery centers and other types of health facilities? Give examples of how this can be improved?***

**Response:** Their work has been well explained to them. Basically, we need their cooperation most of all through the orientation of UHFPO sirs. If the manager is good, everything will work well. You say the midwife, the nurse says everyone will work properly. Orientation is very necessary to minimize. Also monitoring and mentoring is very important. We have seen that where the manager is good, everything is going well.

***27. Question: In midwife-led delivery services, midwives influence effective coordination and partnership among other health professionals involved in maternal and newborn health care, and give examples of how it could be improved?***

**Response:** Certified midwives work where the number of midwives is less. Support staff is very much needed. Most of the nannies. Support staff is very much needed during delivery. Most of what we hear is that madam, we have no support staff, no gatekeepers. They are very lacking at the union level. It is a team work team, everyone has to work together. We also have to look at the security aspect of midwives. DGNM provides midwife but DGHS provides HR. It is very important to have support staff. Supervisors are also very important to point out their mistakes. For the last 10-12 years, only discussions are going on to fix them. , they don't appear to be working implements.

***28. Question: Give examples of how any modern methods or technologies used as part of services provided by midwives could be made useful?***

**Response:** This country does not have advanced equipment in this sector. Foreign countries are much advanced. However, we have recently introduced many new equipment and technologies which have made our work more advanced. Pain management has improved a lot. Labor pain through use of bath tub pain relief balls etc. More improved. Now we should supply minimum equipment at every union level so that good service can be provided everywhere. It should be ensured. It should be continuously monitored. Technology and equipment should be updated day to day. And the number of midwives should be increased. Services should also be provided in remote areas like our Cox’s bazar, Khagrachari, Bandarban area. The government should work more strongly in this regard. The number of HR should be increased. If this profession can be increased, there will be a lot of improvement in the country.

Thank you for giving your valuable time. If we need your help we’ll call you again.
